# Study protocol for a multicenter phase II prospective externally controlled non-inferiority trial of hypofractionated re-irradiation in patients with recurrent high-grade glioma (RISinG)

**DOI:** 10.1371/journal.pone.0342337

**Published:** 2026-02-09

**Authors:** A. M. de Jong, A. T. J. van der Boog, C. C. B. Post, F. Cialdella, D. B. P. Eekers, J. Hendrikse, M. C. A. Kramer, F. J. Lagerwaard, M. van der Meulen, M. E. P. Philippens, P. A. Robe, T. Rozema, F. Y. F. L. de Vos, J. Vos-Westerman, H. L. van der Weide, G. Wester, J. Zindler, T. J. Snijders, J. J. C. Verhoeff

**Affiliations:** 1 Department of Radiation Oncology, University Medical Center Utrecht, Utrecht, the Netherlands; 2 Department of Neurology & Neurosurgery, UMC Utrecht Brain Center, University Medical Center Utrecht, Utrecht, the Netherlands; 3 Department of Radiation Oncology (Maastro), GROW School for Oncology and Reproduction, Maastricht University Medical Centre+, Maastricht, the Netherlands; 4 Department of Radiology, University Medical Center Utrecht, Utrecht, the Netherlands; 5 Department of Radiation Oncology, University Medical Center Groningen, Groningen, the Netherlands; 6 Department of Radiation Oncology, Amsterdam University Medical Center, Amsterdam, the Netherlands; 7 Department of Neurology, Medisch Spectrum Twente, Enschede/ department of Neurology Leiden University Medical Center, Leiden, the Netherlands; 8 Department of Radiation Oncology, Instituut Verbeeten, Tilburg, the Netherlands; 9 Department of Medical Oncology, University Medical Center Utrecht, Utrecht, the Netherlands; 10 Department of Radiation Oncology, Isala Klinieken, Zwolle, the Netherlands; 11 Department of Radiation Oncology, Radiotherapiegroep Arnhem, Apeldoorn, Deventer, Ede, the Netherlands; 12 Department of Radiation Oncology, Haaglanden Medisch Centrum, Den Haag, the Netherlands; PLOS: Public Library of Science, UNITED KINGDOM OF GREAT BRITAIN AND NORTHERN IRELAND

## Abstract

**Background:**

Reirradiation is a widely accepted option for second-line treatment in patients with recurrent glioma. However, no standard radiation regimen has been defined. Hypofractionation is aimed at reducing patients’ burden while maintaining the survival benefit, but may increase the risk of radionecrosis. The primary objective of this study is to determine if reirradiation in just 4 fractions is non-inferior to 10 fractions, regarding survival after reirradiation.

**Methods:**

RISING trial A was an open label, randomized, non-inferiority, phase III trial with 1:1 allocation for 130 patients among all participating centers but failed to recruit according to planning. RISING trial B will be a phase II, multi-center, clinical trial with a historic control group. The experimental group receives 4 stereotactic fractions. The historic control group has received 10 fractions (standard-of-care) with a biologically equivalent dose on surrounding brain tissue. The primary endpoint is overall survival after reirradiation. The key secondary endpoint is progression-free survival. Other secondary endpoints are recurrence patterns, toxicity (specifically clinically relevant radionecrosis) and anti-edema treatment. We will collect and report on Health-Related Quality of Life (HRQoL) data in the experimental arm.

**Discussion:**

We expect to demonstrate the non-inferiority and safety of a 4-fraction hypofractionation schedule for reirradiation of gliomas. This schedule may then become a standard-of-care option with minimal burden for patients with recurrent glioma, and limited use of scarce healthcare resources.

**Trial registration:**

Registered at Netherlands Trial Register (NTR), Trial ID: NL72766.041.20, registered at 07-04-2020, https://www.onderzoekmetmensen.nl/en/trial/52643

## Introduction

### Background and rationale

The diagnosis of high grade glioma is often associated with an extremely poor prognosis, with a median overall survival time (mOS) for glioblastoma patients, ranging from 9 months to 20 months [1,2]. Despite multimodality treatments, the local recurrence rate is high (nearly 90% within 2 years [1,3]). Additionally, primary low grade gliomas will eventually recur as a high grade glioma at a median of 60–84 months after primary treatment, which usually includes surgery, radiotherapy and chemotherapy [4]. Although gliomas are relatively rare, they cause the highest average number of life years lost of all cancers at 20 years, for example compared to prostate cancer at 6.1 years [5].

No standard second-line treatment for this orphan disease has been defined [6,7]. Next to re-resection and chemotherapy, reirradiation can be considered as a safe and effective option, leading to mOS ranging from 8 to 12 months from treatment start [8,9]. However, a standard protocol for reirradiation of glioma does not exist and a large variation of radiation treatments is used, with regard to total dose, size and number of fractions. For example, in The Netherlands, more than ten different reirradiation regimens are used for the 2–6 patients that are treated annually in each individual center (personal communication). This forms a good illustration of the large treatment variability. Within this variability, a commonly used schedule is 35 Gy in 10 fractions of 3.5 Gy (Equivalent Dose in 2 Gray Fractions (EQD2) 48 (α/β = 2)); this schedule has been incorporated in the current ESTRO/EANO guideline [8].

Traditionally, radiotherapy is delivered in several daily fractions to allow a high enough dose without exceeding normal tissue tolerance [10]. In recent years, radiotherapy techniques have been improved, allowing more precisely delivered treatment schedules and hypofractionation [11]. Hypofractionation is a treatment regimen that delivers higher doses of radiation in fewer fractions, resulting in reduced treatment time and less hospitals visits.

In addition, it may achieve increased cell kill from a higher dose per fraction. Nowadays, hypofractionation is frequently used in the treatment of recurrent glioma, appearing to be safe and effective [8,12]. However, there is a lack of studies that compare the effect of different (hypofractionation) regimens directly. To date, research is mostly limited to single center studies with one reirradiation schedule, as summarized in a comprehensive systematic review on reirradiation in glioblastoma [12].

One of these retrospective studies is a retrospective Dutch cohort study, analyzing 121 re-irradiated patients from two centers. This study showed no statistically significant difference in OS between patients treated with conventional fractionated radiotherapy (defined as <3Gy/fraction), hypofractionated radiotherapy (3–5Gy/fraction), or stereotactic radiotherapy (≥5Gy/fraction), even after correcting for Karnofsky performance status (KPS), time interval, planning target volume (PTV) and initial WHO grade [13]. Another single center retrospective cohort study compared schedules with 6 fractions of 5 Gy (EQD2 52,5 (α/β = 2)), 12 fractions of 3 Gy (EQD2 45 (α/β = 2)), and 23 fractions of 2 Gy (EQD2 46 (α/β = 2)) to a schedule with 18 fractions of 2 Gy (EQD2 36 (α/β = 2)) in 199 patients [14]. mOS was 9.6, 6.7, 11.3, and 7.6 months, respectively. The difference between the groups was significant in univariable (p = 0.04) and multivariable analysis (p = 0.02) including the covariates initial WHO grade, age, time interval, KPS, neurological symptoms, PTV, and dose group. This difference was mainly caused by the contrast between the two normofractionated groups (Hazard Ratio [HR] 2.45; 95% confidence interval [CI]: 1.11–5.38) in favor of the higher biological dose of 46 Gy. The heterogeneous dose definitions, tumor- and patient characteristics among studies makes comparison between studies difficult. For these reasons, no standard protocol for reirradiation of gliomas has been defined to date.

Radionecrosis is a well-known complication of (cerebral) radiotherapy, and may cause significant morbidity. In order to determine the optimal reirradiation schedule for recurrent high-grade glioma, the risk of radionecrosis needs to be taken into account.

The higher the biological reirradiation dose, the smaller the healthy tissue volume should be: the limiting factor of the re-irradiation dose is the tolerance dose of healthy brain tissue. According to a study by Sminia et al, the applied total cumulative dose is the most important factor with regard to development of radionecrosis occurring at cumulative Normalized Total Dose (NTD_cumulative_) > 100 Gy [15].

Radionecrosis and other adverse events (AEs) in reirradiation retrospective studies are often poorly registered and therefore underestimated [12]. Toxicity after reirradiation can be categorized as acute, occurring within the first 3 months, or late, manifesting months to years after treatment.

Acute toxicity includes fatigue, headaches, nausea and alopecia and skin erythema. Only few studies reported severe acute toxicity with rates of grade 3–4 toxicity ranging from 7.5% to 10% [14,16,17].

Late toxicity after reirradiation encompasses mainly radionecrosis and neurocognitive deficits. Reported radionecrosis rates vary widely, ranging from approximately 9% to 30% across retrospective studies [16,18], while prospective data have also documented substantial rates of irreversible radiation-induced brain changes [19]. Any true differences in toxicity could be attributed to differences in radiotherapy prescription in EQD2 and patient characteristics. However, the large variation among studies is probably caused primarily by underreporting in retrospective studies. It is of great importance to accurately record and analyze AEs after reirradiation to assess safety and its influence on Health-related Quality of Life (HRQoL).

Thus far, HRQoL measurement has been poorly investigated in the setting of recurrent glioma. Decline in neurocognitive functioning (NCF) is common in the disease trajectory of a glioma patient and is a main contributor to loss of HRQoL [20]. Given the overall poor prognosis and high prevalence of impaired functioning in patients with recurrent glioma, HRQoL is of extreme importance to patients and their caregivers [21]. A regimen with a small number of fractions could be assumed to contribute to better preservation of HRQoL.

These patient-centered outcomes can provide supportive information about the clinical benefit of the therapy and may facilitate patients’ and physicians’ decision-making.

To minimize toxicity of re-irradiation for this palliative treatment setting, radiation oncologists often may be reluctant to irradiate larger tumors (e.g., exceeding 5 cm in diameter). In addition, they typically use small margins to avoid damage to normal brain tissue. However, since the maximum safe reirradiation volume is unknown, this may lead to undertreatment of patients with larger tumors [8]. In the study of Shen et al. with a median reirradiation volume of 202cc (and dose 30–54 Gy in 1.5 to 2.0 Gy fractions), volume was not associated with differences in side effects or survival; these data suggest that larger volume might not be an appropriate exclusion factor for patients who may otherwise be appropriately treated with reirradiation [22]. In the current study protocol the maximum accepted Gross Tumor Volume (GTV) for reirradiation will be 125cc (about 6 cm diameter). This corresponds to the 90^th^ percentile of the patients re-irradiated in UMC Utrecht and UMC Groningen cohorts [13]. Several studies performed reirradiation in gliomas including or beyond this volume. This includes both hypofractionated [19,23–25] and normofractionated regimens [18,26,27]. A clinical target volume (CTV) margin of 1 cm will be used to treat the potentially microscopic tumor cells in this zone. A lower dose administered to this zone could limit the risk of radionecrosis and other adverse events, while a simultaneous integrated boost (SIB) to the contrast enhanced area on T1 MRI may kill tumor cells more effectively.

Therefore, the RISING trial aims to compare a 4 fractions regimen of reirradiation with a 7.5 Gy SIB (experimental arm) to a conventional generally accepted schedule with 10 fractions of 3.5 Gy (control arm) regarding the efficacy and safety of these dose prescription methods in recurrent high-grade glioma with a diameter of up to 6 cm (125cc). The trial was originally designed as a prospective trial (RISING part A) with a 1:1 randomization between 4x7.5 and 10x3.5 Gy schedules, however, insufficient inclusion rates led to a conversion to the current design (RISING part B): a phase II trial with a prospective experimental arm of 4x7.5 Gy, compared to a historical control group of 10x3.5 Gy.

## Objectives

The primary objective is to determine if reirradiation in 4 fractions is non-inferior to 10 fractions, regarding overall survival after reirradiation. Only if non-inferiority is demonstrated, superiority will be tested (hierarchical design).

Secondary objective(s) include:

1. To compare progression-free survival (PFS) between the two treatment groups.
2. To describe HRQoL in the experimental treatment group
3. To assess NCF in a subgroup of the experimental treatment group (RISinG trial A only).
4. To describe toxicity in the experimental treatment group in detail, and compare grade ≥3 toxicity in both groups.
5. To establish and compare recurrence patterns on MRI between the two treatment groups.

## Materials and methods

### Trial design

The original 2019 RISING-trial (RISING trial A) was designed as an open label, randomized, non-inferiority, phase III trial with 1:1 allocation for 130 patients. From 2019 until 2023, 13 patients were included in the experimental arm and 16 patients were included in the control arm of RISING trial A. Due to this poor accrual and concerns about study completion (and availability of sufficient funding to achieve completion), the steering committee amended the trial design. The updated 2023 RISinG trial (RISING trial B) is designed as a phase II, multi-center, prospective clinical trial with a historical control group. The additional recruitment period of this phase will be approximately 30 months with an active follow-up time of 12 months.

The 13 patients of the RISING trial A will be supplemented until a total of 66 patients is reached. These patients fulfilling the inclusion criteria will get the following treatment:

- Hypofractionated radiotherapy with SIB in 2 weeks

4 fractions of 7.5 Gy SIB to the PTV_SIB_ (GTV + institution-specific margin (based on local equipment and protocols))
And 4 fractions of 5.5 Gy to the PTV (GTV + 5 mm margin).

We will compare the survival of this group with a retrospective control group of patients, selected from the retro-RISinG study. Retro-RISinG is a multi-center retrospective registry study, in which details of patient-, tumor- and treatment-related data of consecutive patients who received reirradiation for recurrence of a diffuse glioma since January 2002 up to December 2022 are collected. This study encompasses the majority of radiation oncology centers in The Netherlands. We will select 99 matched patients in total with the following treatment:

- Moderately hypofractionated radiotherapy in 2–3 weeks

10 fractions of 3.5 Gy to the PTV (CTV + institution-specific margin).

An overview of the target volume definitions and dose prescriptions is given in Table 1.

**Table 1:** Overview of target volume definitions and dose prescriptions.

	Experimental treatment group 4 x 5.5 Gy with 7.5 Gy SIB
**GTV**	Contrast enhancement on T1 MRI
**CTV**_**30Gy (boost)**_	GTV
**CTV**_**22Gy**_	GTV + 5 mm
**PTV**_**30Gy (boost)**_	CTV_**30Gy (boost)**_ + 2 or 3 mm
**PTV**_**22Gy**_	CTV_**22Gy**_ + 2 or 3 mm
**Accepted dose heterogeneity PTV**_**30Gy (boost)**_	95% − 107% D_max 0.1cc_
**Accepted dose heterogeneity PTV**_**22Gy**_	95% − 130% (exclude PTV_30Gy_) D_max 0.1cc_

This control group will be matched on a group level based on propensity score to the patients in the experimental treatment group on an individual level for:

- Age;
- Gender;
- WHO-grade of the primary tumor (according to WHO 2021 classification);
- Time interval in between last fraction of first radiation and first fraction of reirradiation;
- MGMT-status (only for glioblastoma, IDH-wildtype, WHO grade 4).

#### Patient involvement.

Patients and public representatives were involved in the design of the trial. The patient platform of the Dutch Neuro-Oncology working group (LWNO) and the existing neuro-oncology advisory panel at the UMC Utrecht provided input during study development, and their feedback was incorporated into the study documents. Throughout the conduct of the trial, patients will continue to be engaged through these platforms, including regular updates on study progress and opportunities to provide feedback. Regular meetings are planned to facilitate communication and co-creation.

### Participants, interventions and outcomes

#### Trial setting.

In this phase II trial, a multicenter approach is employed, encompassing the participation of several research centers, both academic hospitals as well as community hospitals in the Netherlands. The participating centers are: University Medical Center Utrecht, Maastro (Radiation Therapy and Research Center), Amsterdam University Medical Center, Radiotherapy Group Arnhem/Deventer, Haaglanden Medical Center, Verbeeten Institute Tilburg, Isala Klinieken Zwolle, Medical Spectrum Twente, and University Medical Center Groningen.

#### Eligibility criteria.

In order to be eligible to participate in study group 1 (prospective, experimental group) and 2 (retrospective, control group), a subject must meet all of the following criteria:

- Supratentorial recurrent grade 3 and 4 glioma with contrast enhancement on contrast-enhanced T1-weighted imaging (CE-T1 MRI). In cases of an original grade 2 diagnosis, patients may be included in case of clear (radiological and/or histological) progression to a high-grade lesion, as determined by the local investigator.
- While the gold standard is histological evidence of a recurrence, a recurrence may be diagnosed by radiological imaging alone, if the following conditions are met:
  - ◦ Surgery acquiring histological evidence of recurrence is not feasible.
  - ◦ Agreement of the tumor board and/or a consultant neuro-radiologist that imaging changes are in keeping with recurrence.
  - ◦ Radiological changes meet the Response Assessment in Neuro-Oncology (RANO)criteria for tumor progression [28].
  - ◦ If needed, additional imaging sequences such as PET and perfusion MRI support the diagnosis or recurrence/progression.
- Unifocal glioma (i.e., lesions clustering around residual surgical cavity).
- Prior course of treatment including radiotherapy with an EQD2 (α/β = 2) of at least 47Gy.
- Age ≥ 18 years.
- KPS 60 or above.

For group 1 only, the following additional conditions must be met:

- Ability of subject to understand nature and individual consequences of the clinical trial
- Eligible for treatment, based on the MRI results (e.g., T1-CE diameter >6 cm, reflecting a spherical tumor of 125cc)

For group 2 only, the following additional conditions must be met:

- Patient underwent reirradiation with a treatment schedule of 10x3.5 Gy.

A potential subject who meets any of the following criteria will be excluded from participation in this study (treatment group 1 and 2):

- Previous reirradiation or prior radiosurgery or prior treatment with interstitial radioactive seeds.
- CE-T1 MRI tumor diameter greater than 6 cm (reflecting a spherical tumor of more than 125cc).
- Time interval of less than 6 months after prior radiotherapy.
- Time interval of less than 3 weeks after last re-resection (1 week for biopsy).
- Known malignancy < 3 years ago (excluding carcinoma in situ of the cervix, basal cell carcinoma, squamous cell carcinoma of the skin) requiring immediate treatment and/or interfering with study therapy.
- Women of childbearing potential without adequate contraception.

Screen-failures for treatment group 1 will be replaced by a new patient. These screen-failures are not included in the statistical analysis of the trial and treated with the physicians’ choice.

#### Intervention and comparator.

Radiotherapy will be planned and delivered in every participating center according to the treatment protocol.

##### Experimental treatment group (group 1):

Since prospective phase III research on the optimal fractionation reirradiation regimen is sparse, a radiobiological theoretical optimal regimen is chosen from retrospective studies and phase I/II studies [8,23,24,29,30]. The linear-quadratic model describes cell killing, both for tumor control and for normal tissue complications following exposure to a varying amount of radiation. The most modern method of comparing fractionation schedules is to use the linear quadratic equation to calculated the equivalent dose in 2 Gy fractions (EQD2).

For GTV with a 5 mm margin (CTV_22Gy_), the EQD2 may be up to 40 Gy based on the maximal cumulative dose of 100 Gy advised by Sminia and Mayer [15,31]. For this reason, the dose was selected to be 4 fractions of 5.5 Gy (EQD2_α/β=2_ = 41 Gy).

For the tumor (CE on CE-T1 MRI, GTV), the maximal dose should not exceed 8 fractions of 5 Gy (EQD2_α/β=2_ = 70 Gy). In a prospective phase I trial, hypofractionated stereotactic radiotherapy was given with 5 Gy per fraction to doses ranging from 20 to 50 Gy on a dose escalation program, in patients with recurrent glioma with a median target volume of 24cc. Exceeding 8 fractions of 5 Gy resulted in 6.4 times more radiation damage [32]. Therefore, four fractions of 7.5 Gy were considered safe with a comparable EQD2 _α/β=2_ of 71 Gy.

Experimental treatment schedule therefore consists of 4 fractions of 7.5 Gy SIB to the CE on CE-T1 MRI and 4 fractions of 5.5 Gy to the GTV with a 5 mm margin.

##### Standard treatment group (historic controls, group 2):

In 1999, a phase I dose escalation of hypofractionated stereotactic radiotherapy in 20 patients with recurrent or persistent malignant gliomas was conducted. Three different total dose levels were sequentially evaluated: 24.0 Gy in 8 fractions of 3.0 Gy, 30.0 Gy in 10 fractions of 3.0 Gy and 35.0 Gy in 10 fractions of 3.5 Gy. No grade 3 toxicities were observed and the response rate was 78% [33]. From that time, 10 fractions of 3.5 Gy is a generally accepted regimen. Until now, this schedule has shown to be safe and effective in practice [34]. EQD2_α/β=2_ of this schedule is 48 Gy.

##### Equipment and planning:

Radiotherapy will be planned and delivered with modern techniques including at least intensity modulated radiation therapy (IMRT) and volumetric modulated arc therapy (VMAT).

##### Immobilization:

All participants will be immobilized in a customized thermoplastic shell or relocatable stereotactic frame in a supine position according to institutional standards.

##### Data acquisition and definitions of target volumes and organs at risk:

The definition of volumes will be in accordance with the International Commission on Radiation Units and Measurements (ICRU) Report 50, ICRU Report 62. Volumes will be defined based on a volume MRI scan with contrast (T1 + Gadolinium, CE-T1) and co-registered to a computerized tomography (CT) scan. The MRI scans must have a maximum 2 mm and isotropic voxels, the CT should have 1–3 mm slice thickness. Amino-Acid-PET or SPECT-Examinations may be used in addition to contrast-enhancement on MRI for target volume definition but are not mandatory.

##### Gross Tumour Volume (GTV):

The GTV is defined as the contrast enhancing lesion on CE-T1 MRI sequence for both treatment groups. GTV can be expanded using the other clinically acquired MRI sequences according to the expertise of the local radiation oncologist and the medical team.

##### Clinical Target Volume (CTV):

The CTV of both, the experimental treatment group and the standard treatment group, is defined as the GTV with addition of a 3-dimensional margin of 5 mm limited by anatomic borders. The stereotactic CTV_boost_ of the experimental treatment group is equivalent to the GTV. CTV can be expanded using the other clinically acquired MRI sequences according to the expertise of the local radiation oncologist and the medical team.

##### Planning Target Volume (PTV):

The PTV and PTV_boost_ are defined as the CTV and stereotactic CTV_boost_ plus an isotropic 2 or 3 mm margin depending on radiotherapy center, to account for day to day setup variation related to the ability to immobilize the participant. The PTV should not extend outside the body contour.

##### Organs at Risk (OARs):

OARs include the following normal tissue volumes: the lenses, optic nerves and chiasm and the eyeball (includes anterior and posterior chambers); brainstem; pituitary; cochlea; skin (a 3 mm thick rim) and the uninvolved brain (= brain – CTV). OARs will be delineated following the local guidelines of the participating centers and will typically follow atlases such as CT-based OAR atlas [35] or the European Partical Therapy Network (EPTN)-consensus based atlas [36]. The complete atlas is provided as supplemental material of the published article, available at https://ars.els-cdn.com/content/image/1-s2.0-S0167814015004016-mmc1.pdf.

##### Recovery potential:

With regard to the recovery potential in the brain after prior irradiation, the following schedule will be utilized [37]:

- No recovery within 1 year.
- 30% recovery after 1 year.
- 50% recovery after 2 years.

##### Treatment planning:

IMRT or VMAT may be planned using fixed fields or arcs. The treatment plan to be used for each participant is based on an analysis of volumetric doses including the dose volume histogram (DVH) of the PTV and the OARs. Treatment planning should conform to ICRU 50, 62 and 83 rules for coverage of GTV, CTV and PTV. OAR planning risk volumes (PRV) should be defined by applying a margin equal to the PTV margin.

##### Prescribed dose and fractionation:

The dose prescribed to the PTV in the experimental group is 4 fractions of 5.5 Gy. The prescribed dose to the PTV_boost_ is 4 fractions of 7.5 Gy. All fractions should be given within 2 weeks. The maximum dose in the PTV_boost_ of the experimental group should not exceed 107% of the prescription dose.

The maximum dose in the PTV (i.e., PTV minus PTV_boost_) should not exceed 130% of the prescription dose according to ICRU rapport 50. The dose calculation grid should be 3 mm or less.

##### Dose limitation to organs at risk:

It is important to minimize the dose to the OARs whenever possible. This must be weighed against the possibility of sub-optimal treatment of the target volume. The dose constraints for the Organs at Risk are given in Table 2.

**Table 2:** Dose constraints for OARs in Gy. Radiobiological calculations performed with α/β = 2Gy. To calculate the dose constraint at reirradiation, the initial dose should be subtracted from the cumulative dose constraint for OARs. Subsequently, the reirradiation EQD2 should be converted to the dose constraint for the OARs according to the treatment group. The reirradiation dose of OARs should never exceed the EQD2 and total dose constraint given for initial treatment (shown in column ‘Constraint at reirradiation’).

Constraint	OAR	Constraint at reirradiation (without considering previous radiation therapy)		Cumulative constraint (Initial RT + reirradiation)
Total D_max 0.1cc_ dose (unless otherwise specified) at **α/β = 2**		
EQD2	Total physical dose at 4 x 7.5 Gy and 4 x 5.5 Gy	EQD2
Hard	Optic nerves	≤ 60 Gy	≤ 27 Gy	≤ 90 Gy
	Optic chiasm	≤ 60 Gy	≤ 27 Gy	≤ 90 Gy
	Brain stem	≤ 60 Gy	≤ 27 Gy	≤ 90 Gy
Soft	Optic nerves	≤ 55 Gy	≤ 26 Gy	≤ 83 Gy
	Optic chiasm	≤ 55 Gy	≤ 26 Gy	≤ 83 Gy
	Brain stem	≤ 55 Gy OR 54-59 Gy < 10cc	≤ 26 Gy OR 26-27 Gy < 10cc	≤ 83 Gy OR 81-89 Gy < 10 cc
	Eye balls	≤ 45	≤ 23 Gy	≤ 68 Gy
	Lenses	D_Mean_ ≤ 6 Gy D_max_ 10 Gy	D_Mean_ ≤ 6 Gy D_max 0.1cc_ 9 Gy	D_Mean_ ≤ 9 Gy D_max 0.1cc_ 15 Gy
	Pituitary Gland	D_Mean_ ≤ 45 Gy	D_Mean_ ≤ 23 Gy	D_Mean_ ≤ 68 Gy
	Cochlea	D_Mean_ ≤ 45 Gy	D_Mean_ ≤ 23 Gy	D_Mean_ ≤ 68 Gy

*Example*: Optic Chiasm reirradiation dose = 9.8Gy (≤ 38) in 10 fractions = 7Gy EQD2 (≤ 54). Initial dose 53.2Gy in 30 fractions = 51Gy EQD2. Cumulative dose = 51 + 7 = 58Gy, is ≤ 81.

##### Timing of radiotherapy:

The interval between the planning-MRI and actual first fraction of reirradiation is 3 weeks at most.

##### Criteria for discontinuing or modifying allocated interventions:

Subjects can leave the study treatment group at any time, for any reason, if they wish to do so, without any consequences. The investigator can decide to withdraw a subject from the study for urgent medical reasons. If a patient is going off protocol treatment, the reason should be documented. Patients are not obligated to specify the reason of withdrawal.

##### Strategies to improve adherence to interventions:

Participants are actively encouraged to attend all scheduled radiation therapy sessions to ensure adherence to the study protocol.

Attendance will be monitored, and participants will be contacted to reschedule any missed appointments in a timely manner. Participants are encouraged to complete and return any questionnaires sent to them within the specified timeframe. If a questionnaire has not been completed within two weeks, a reminder will be sent to encourage timely submission.

##### Relevant concomitant care permitted or prohibited during the trial:

Concomitant dexamethasone and anticonvulsant therapy is allowed, in line with routine clinical care and local/national guidelines. Salvage surgery prior to start radiation is allowed. Concurrent systemic therapy including chemotherapy, immunotherapy or targeted therapy during reirradiation is not allowed, including the use of bevacizumab.

#### Outcomes.

The primary endpoint will be the time to death (overall survival (OS)) counted from the first fraction of reirradiation until death due to any cause. Both treatments are finished within 2–3 weeks.

The key secondary endpoint is:

- Progression free survival (PFS) counted from the first date of reirradiation until the date of MRI evidence of tumor recurrence or death due to any cause. Agreement of the tumor board or a consultant neuro-radiologist that imaging changes are in keeping with recurrence and not radionecrosis is necessary.

Other secondary endpoints include the following:

- Neurocognitive function (NCF) (RISinG trial A only)
- Recurrence Patterns after reirradiation on MRI.
- Radiation toxicity graded by CTCAE v5.0, including radionecrosis (group 1 only due to limited toxicity data availability in group 2)
- Use of anti-edema therapy (including Dexamethasone and Bevacizumab use).
- HRQoL prospectively monitored by EQ-5D-5L, EORTC QLQ-C15-PAL and EORTC-QLQ-BN20 + 2 questionnaires (group 1 only).

The chosen endpoints are critical for evaluating both the effectiveness and safety of the reirradiation treatment.

#### Harms.

All adverse events will be registered prospectively in treatment arm 1. For the retrospective control arm (arm 2), AE’s will be extracted from electronic patient files in a similar manner.

##### Adverse events (AEs):

Adverse events are defined as any undesirable experience occurring to a subject during the study, whether or not considered related to reirradiation treatment. All adverse events reported spontaneously by the subject or observed by the investigator or his staff will be recorded.

##### Serious adverse events (SAEs):

A serious adverse event is any untoward medical occurrence or effect that:

- results in death.
- is life threatening (at the time of the event).
- requires hospitalization or prolongation of existing inpatients’ hospitalization.
- results in persistent or significant disability or incapacity.
- is a congenital anomaly or birth defect.
- any other important medical event that did not result in any of the outcomes listed above due to medical or surgical intervention but could have been based upon appropriate judgment by the investigator. An elective hospital admission will not be considered as a serious adverse event.

The investigator will report all SAEs to the sponsor without undue delay after obtaining knowledge of the events. However, patients with recurrent glioma have a poor life expectancy. Therefore, a significant proportion of patients will die within the study duration of 1 year after radiotherapy. The SAE sensitive period will be up until 30 days after last reirradiation fraction to account for treatment related effects. Expected SAEs are hospitalization, persistent or significant disability or incapacity, or death due to glioma progression. These expected SAEs will be reported every year in a line listing.

The sponsor will report the SAEs through the web portal ToetsingOnline to the accredited METC that approved the protocol, within 7 days of first knowledge for SAEs that result in death or are life threatening followed by a period of maximum of 8 days to complete the initial preliminary report. All other SAEs will be reported within a period of maximum 15 days after the sponsor has first knowledge of the serious adverse events.

##### Follow-up of adverse events:

All AEs will be followed until they have abated, or until a stable situation has been reached. Depending on the event, follow up may require additional tests or medical procedures as indicated, and/or referral to the general physician or a medical specialist.

#### Participant timeline.

**Fig 1:**
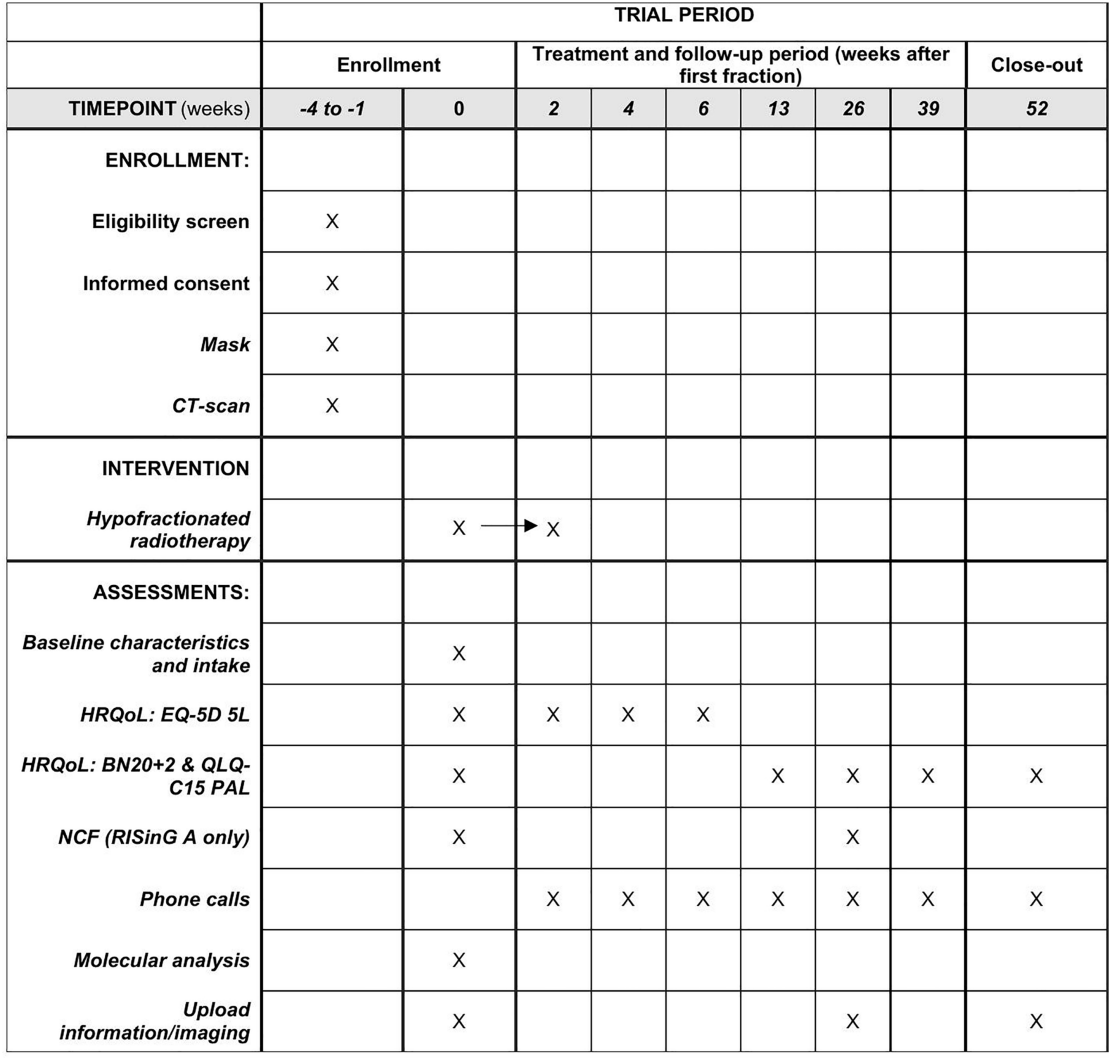
Participant timeline: Schedule of enrollment, interventions and assessments.

Fig 1 provides a detailed schedule of enrollment, interventions and further assessment throughout the study period.

Participant timeline, summarizing the timing of key study activities, including eligibility screening, informed consent, imaging, hypofractionated radiotherapy and study assessments, including health-related quality of life (HRQoL), neurocognitive functioning (NCF), molecular analysis and scheduled phone calls for follow-up. Used questionnaires include EQ-5D 5L (EuroQoL five-dimension five-level Questionnaire, QLQ-C15 PAL (EORTC Quality of Life Questionnaire Core 15 Palliative care), BN20 + 2 (EORTC Brain Neoplasm questionnaire with 2 additional questions).

#### Sample size.

The sample size required to achieve a power of 1-β = 0.80 for the one-sided chi-square test at level α = 0.05 when the hazard ratio is actually 0.90, and a distribution of 40% in the intervention group and 60% and the control group, amounts to 165 subjects: 99 in the control group and 66 in the treatment group [38] The non-inferiority ratio is 1.4. Unfortunately, there are no historical randomized placebo-controlled trials for treatment group 2 to use to formulate a margin, therefore we collect this data now as well for matched patients. This ratio is based on clinical judgment of the calculated survival fractions at the proposed non-inferiority margins and published survival data [13,39].

To clarify the meaning of this non-inferiority margin in practice, the non-inferiority margin specific survival fraction at median survival time could be calculated. This non-inferiority margin means in practice that at least 76% of the patients surviving at the median survival time of the control group would have survived when treated with the experimental treatment. In other words, at least 38 percent of the patients in the intervention group reach the median survival time instead of 50 percent in the control group.

Expected event rate at the minimal follow-up time of 12 months is 0.7 based on the literature [13,39]. However, a part of the study population may have a longer follow-up period of even up to 3.5 years.

It is anticipated that the proportion of subjects observed with the event during the total study period is 0.8. These results assume that the hazard ratio is constant throughout the study and that Cox proportional hazards regression or the non-inferiority log rank test is used to analyze the data. To compensate for any drop-outs or slight deviation from the assumptions made, the total sample size should be 165 patients.

A point of discussion is that we anticipated that the hazard ratio is actually 0.90, however we can in fact not know the actual hazard ratio. It is justified to assume a hazard ratio below 1.0 when hypothesizing that the tumor will be treated slightly better by a higher dose per fraction.

The trial is primarily designed to show non-inferiority, in addition superiority will be tested. If the actual hazard ratio is lower, the patient number will be sufficient to detect superiority.

#### Recruitment.

Participants will be actively screened through multidisciplinary boards at each participating center.

Regular newsletters will be sent to all participating centers to keep them informed about the study and any updates regarding new patient recruitment. These newsletters will include information on the study’s progress, eligibility criteria, and any other relevant updates.

The study will be discussed regularly in the Dutch Working group Neuro-oncology (LWNO) and the Dutch national platform for neurological radiation oncology (LPRNO). To improve inclusion compared to RISinG trial A, we included more centers and we took several measures to maximize inclusion in each participating center.

### Data collection, management and analysis

#### Data collection methods.

##### Plans for assessment and collection of outcomes:

The clinical data from the experimental arm will be collected from medical charts, pathology reports, radiology reports and web-based surveys (PROFILES web-based database). The data will be entered and managed using Castor EDC (Castor, Amsterdam, The Netherlands), a secure electronic data capture system.

Health related quality of life (HRQoL)

Patients will complete HRQoL questionnaires and the study team will record toxicity and anti-edema treatment by phone call at baseline and at 2, 4, 6, 13, 26, 39, and 52 weeks from the start of reirradiation or until disease progression (whichever is sooner). During the first 6 weeks HRQoL will be assessed with use of the EQ-5D-5L. At subsequent time points QLQ-C15-PAL and BN20 + 2 will be completed digitally or on paper.

EQ-5D-5L is a validated measure of health status developed by the EuroQol Group in order to provide a simple, generic measure of health for clinical and economic appraisal [40,41]. This tool is used for the HRQoL measurement during the first 6 weeks after therapy start, since it takes only a few minutes to complete.

Subsequently, HRQoL will be determined using the EORTC QLQ-C15 PAL questionnaire [42] and QLQ-BN20 + 2 questionnaire [43] every 3 months after finishing radiotherapy treatment. The QLQ-C15 PAL is a validated general core QoL assessment tool which is developed for (near) palliative cancer care patients. The QLQ-BN20 + 2 includes the QLQ-BN20 questionnaire that has been validated for use in patients with primary brain malignancies. In addition, two scales for concentration and memory were added which were omitted in the abbreviated QLQ-C15-PAL.

The QLQBN20 + 2 was first described by Caissie et al. and validated by Nguyen et al. in patients with brain metastases [43,44]. These questionnaires consist in total of 37 assessments instead of 50 assessments when using the QLQ-C30. This aims to minimize patients’ burden and increase the chance of maintaining follow-up as patients progress through their disease course [43].

Neurocognitive assessment (NCA, RISinG trial A only)

NCA will be measured by a core set of cognitive tests proposed by RANO working groups and International Cognition and Cancer Task Force [44,45]. The tests include measures of memory, processing speed, executive function and verbal fluency. The test battery takes about 30–45 minutes. This core assessment will take place at baseline and after 6 months, and will be administered by trained employees.

##### Plans to promote participant retention and complete follow-up:

Follow-up data will be collected from routine clinical data wherever possible, and do not require specific efforts from patients. If a study-specific questionnaire has not been completed within two weeks, a reminder will be sent to encourage timely submission.

#### Data management and confidentiality.

The handling of personal data will comply with the General Data Protection Regulation (GDPR) (In Dutch: Algemene Verordening Gegevensbescherming (AVG)), so data will be handled confidentially and coded.

Study sites will register a patient in the web-based application Ldot (Ldot, MEMIC, Maastricht University) after informed consent of the patient. Data collected through web-based surveys will be sent to the central RISinG database coded without patient identifiers. Enrolled participants will be assigned study identification codes that will not resemble medical record number, governmental identification or other unique identifiers. Participants who are not able to use the web-based surveys will be offered hardcopy questionnaires instead.

In addition to the pseudonymized clinical data, technical data on imaging and radiation plans will be collected as well. These will be stored in the Research Imaging Architecture (RIA), a fully secure data storage infrastructure.

A separate identification log only containing codes with corresponding patient identifiers (name, date of birth, sex and date of inclusion), will be stored on a different secured location within the UMCU to prevent the possibility to directly match study data with patient identifiers.

#### Statistical methods.

##### Statistical methods for primary and secondary outcomes:

Demographic and baseline characteristics

Data on demographic and baseline characteristics will be summarized by means of descriptive statistics. For continuous variables, in case of normal distribution by means and standard deviations, and in case of non-normal distribution by medians and interquartile ranges. For discrete variables data will be summarized by frequencies and proportions.

OS and PFS

All statistical tests for the efficacy endpoint OS and PFS will be performed according to intention-to-treat (ITT) principle, which is the most unbiased method. Considering the trial being a non-inferiority trial, which is more sensitive to bias, a secondary per-protocol (PP) analysis will be done for OS [46].

The PP analyses will be performed on a “subset of patients who complied sufficiently with the protocol, such as exposure to treatment, availability of measures and absence of major protocol violations” according to the ICH E9 guidelines. The ITT analysis includes all participants that meet the inclusion criteria. Start date of reirradiation will be used as t = 0. Patients not known to have died during the study will be censored for OS at the day they were last known to be alive. Patients free from progression and alive at the last visit will be censored for PFS at the day of last assessment. Endpoints are measured from the first date of reirradiation.

Distributions of OS and PFS will be estimated by the Kaplan-Meier method. The OS rates at one year, PFS rates at six months and medians of OS and PFS will be presented with two-sided 95% confidence interval(CI) computed using the log-log transformation.

To minimize the effect of confounding when estimating the difference between the treatment and retrospective control group, stabilized inverse probability of treatment weighting (SIPTW) using propensity scores will be applied when comparing the groups on OS and PFS.

Propensity scores will be estimated using logistic regression including important covariates for OS and PFS: primary histology, GTV, KPS and age.

OS

The hazard rate for the experimental versus the retrospective control group and its two-sided 80 and 95% CIs will be estimated by means of a Cox proportional hazards model. We will adjust for confounding using propensity scores. We will conclude that 4 fractions is non-inferior to 10 fractions of RT if the higher 90% CI of the hazard ratio (HR) of 4 fractions versus 10 fractions was not above 1.4.

Only when non-inferiority has been demonstrated, we will test for superiority of the experimental treatment in a hierarchical fashion, using Kaplan-Meier method and two-sided log-rank test at a significance level α = 10% in ITT analysis.

PFS

The primary comparison of the time-to-event distributions between the two treatment groups will consist of the two-sided log-rank test at significance level α = 5% The hazard rates for the experimental versus the retrospective control group and its two-sided 95% CI will be estimated with a Cox proportional hazards model. We will adjust for confounding using propensity scores.

Recurrence patterns

Recurrence patterns will be compared between the treatment groups with logistic regression, also adjusted for confounding using propensity scores. Recurrences are defined as “central” if more than 95% of the tumor recurrence resides within the prescription 95% isodose surface (D95) of the PTV; “in-field” if more than 80% of recurrent lesion is inside D95 of the PTV, and “marginal” if 20% to 80% of the lesion is inside D95 of the PTV. In all other cases, recurrences are defined as outside the radiation field (“ex-field”) according to the study of Lee et al [47].

HRQoL in treatment group 1

HRQoL will only be assessed in the experimental group. Reasons for missing baseline and follow-up questionnaires will be assessed. Linear Mixed Effects models will be used for regression analyses of HRQoL. In addition, the repeated measures of the EQ-5D-5L, QLQ-C15-PAL and BN20 + 2 functional and symptom scales will be assessed by time to deterioration (TTD) analyses. The time until definitive HRQoL score deterioration (TUDD) is defined as time from inclusion to a first occurrence of a deterioration of the score that exceeds the minimal clinically important difference (MCID) of at least 10 points compared to the baseline score. This deterioration is considered definitive if there is no subsequent improvement exceeding the MCID relative to the baseline, or if the patient drops out after the deterioration, resulting in missing data. According to the construction of the HRQoL scores, deterioration corresponds to an increase (e.g., for symptomatic scales of the EORTC questionnaires and EQ-5D-5L) or a decrease (e.g., for functional scales) of the score.

Patients with no score available are excluded from the time-to-deterioration analyses. Patients with no baseline score are usually censored at baseline and those with no follow up scores but with a baseline score are censored one day after baseline. Patients with no deterioration before drop-out from the study, including death, are censored at the time of last HRQoL questionnaire completion or last follow-up.

The TUDD estimation will be calculated using the Kaplan-Meier method and described using median and 95% CI.

Neurocognitive Function (RISinG trial A only)

Analyses on both frequency and extent of cognitive decline in both arms will be performed using logistic and linear regression.

Toxicity

Toxicity will only be assessed in the experimental group. Analyses of radiotherapy toxicity will be performed in all patients who received at least one fraction. Acute events occurring from start of radiotherapy until 90 days and late events occurring from 91 days after end of radiotherapy will be presented. Further analyses of treatment toxicity will present the grade 3–5 acute or late side effect by treatment group. The time to occurrence of any severe late side effects, and radionecrosis separately (distinguishing symptomatic and asymptomatic), will be estimated by cumulative incidence. The time to severe late side effects will be calculated from the time of start of radiation treatment to the first evidence of any grade 3–5 late side effects. Patients alive without grade 3–5 late toxicity will be censored at the date of last follow-up, patients who died without experiencing late grade 3–5 side effects will be assessed as competing risk at the time of death.

Prognostic and predictive factors

Additional exploratory analyses using Cox regression analyses will study the prognostic impact of factors (other than treatment, age, gender, WHO-grade primary tumor, time interval in between first radiation and reirradiation and MGMT-status), including baseline KPS, number of recurrence, tumor volume and dexamethasone use at baseline. In addition, the independent predictive value for the treatment effect of the 4 and 10 fraction regimens of these factors will be investigated using Cox Regression analyses.

##### Handling of missing data:

An inclusion of 66 subjects in the experimental arm and 99 in the control group are aimed to generate a total of 165 patients If a subject in arm 1 discontinues from the study prematurely the reason, if given, must be fully evaluated and recorded appropriately.

After informed consent, patients who did not receive any reirradiation (e.g., withdrawal of consent or due to findings on planning-MRI) will be substituted by additional patients. Patients who received at least 1 fraction, withdrawing because of any reason will not be substituted by additional patients.

By only including patients who received at least one fraction of reirradiation in the experimental arm, we aim to optimize comparability with the retrospective control-arm (wherein all patients received reirradiation).

Patients withdrawn from the trial retain their identification codes, if already given. New patients must always be allotted a new identification code. Patients withdrawn from treatment will be asked if they will continue completing the questionnaires and if their medical record may be checked for reported toxicity and progression. Patients will be asked for oral and written informed consent.

Patients withdrawn from treatment will be asked if they will continue completing the questionnaires and if their medical record may be checked for reported toxicity and progression. Patients will be asked for oral and written informed consent.

Due to the possibility of checking the date of death in the National population registry in the Netherlands and due to the fact that reirradiation schedule is one of the inclusion criteria, we do not expect missing data for our primary analysis.

For HRQoL we use a linear mixed model. This method is robust, even in the case of missing data [48].

### Monitoring

#### Data monitoring committee.

Due to the well-known toxicity profile of reirradiation for high-grade gliomas and the low risk of unexpected adverse events, a data safety monitoring board (DSMB) will not be established.

#### Interim analysis.

Six months after inclusion of every 10 patients an interim analysis will be performed to review the toxicity data. If the toxicity is unexpectedly high in large tumors (65–125cc), the maximal tumor diameter will be modified to 5 cm (reflecting a spherical tumor of 65 cc).The detailed interim analyses are not made available to trial participants.

Sequential boundaries will be used to monitor severe toxicity (CTCAE grade ≥3)Full follow-up for acute toxicity is defined as 90 days after last irradiation fraction. Full follow-up for radionecrosis is defined as after the MRI at 6 months.

This is a Pocock-type stopping boundary that yields the probability of crossing the boundary at most 0.05 when the rate of severe toxicity is equal to the acceptable rate 30, 20 or 10% depending on adverse event grading [45].

The research team will be responsible for the interim analysis. The accrual of patients with a tumor with a diameter >5 cm will be halted if excessive numbers of severe toxicities are seen, that is, if the number of severe toxicities is equal to or exceeds b_n_ out of n patients with full follow-up.

No more interim analyses will be performed if accrual of patients with a tumor volume of >5 cm (reflecting a spherical tumor of 65 cc) is halted based on the above, if after 4 iterations of interim analyses, no excessive numbers of severe toxicities are seen, as mentioned above or when inclusion is completed without excessive dose-limiting toxicities. In any of these cases, the Medical Ethics Committee that gave a favorable opinion will be notified.

The most objective toxicity terms will be evaluated, including central nervous system necrosis, edema and seizures. Maximal accepted percentage grade 3–5 radiotherapy related acute toxicity or late radionecrosis (severe symptoms; medical intervention indicated) is 30%. Maximal accepted percentage grade 4 or 5 adverse events is 20%. Maximal accepted percentage grade 5 adverse events is 10%. Toxicity terms are graded according to the CTCAE v5.0. Edema will be graded similar to radionecrosis.

If maximal tumor diameter has to be modified to 5 cm, expected loss of recruitment amounts 15% based on the retrospective dataset of UMC Utrecht and UMC Groningen. The recruitment time should be prolonged with approximately 5 months to achieve a total of 66 patients.

#### Oversight and monitoring.

##### Composition of the coordinating center and trial steering committee:

The main team of the coordinating center is composed of the project controller, one principal investigator and one coordinating investigator. They meet biweekly and discuss day to day issues, concerning trial management, communication with the centers and protocol implementation. The steering committee consists of the main team of the coordinating center, the other members of the writing committee and the trial agency of the division. The principal investigators of the study sites coordinate the execution of the trial in their own center. Together with the steering committee, they are the investigator group. For independent patient support, an independent advisory physician is available.

The monitoring of the study is performed by a qualified and trained monitor to assure the quality and validity of the research data. Due to the well-known toxicity profile of reirradiation for high-grade gliomas and the low risk of unexpected adverse events, a data safety monitoring board (DSMB) will not be established.

##### Study monitoring:

The study will be monitored, by using a minimal, risk-based, primarily on-site monitoring approach, performed by a qualified and trained monitor to assure the quality and validity of the research data. Monitoring activities include review of patient flow, essential documents, informed consent, eligibility and targeted source data verification (minimum 5%), as well as verification of Serious Adverse Event Reporting. An initiation visit will be performed before enrolment, the first routine visit after the first three participants, followed by annual visits per site, with frequency adjusted based on recruitment. A close-out visit will be conducted at study end. Further details are provided in the Monitoring plan.

### Ethics

#### Research ethics approval.

The study protocol was approved by the Medical Ethics Review Committee (METC) NedMec. The first version of the protocol was approved on 7 April 2020, with the latest amendment approved on 13 June 2024. The study is registered under research dossier number NL72766.041.20, version 06, and the METC protocol number is 20–056/A. It can be accessed on the following website: https://www.onderzoekmetmensen.nl/en/trial/52643.

Written, informed consent to participate will be obtained from all participants before any study procedures are conducted.

#### Protocol amendments.

In the event of an important protocol modification, such as changes to eligibility criteria, outcomes and analyses, the updated protocol will first be reported to the Medical Ethics Review Committee ‘NedMec’. After approval, the updated protocol will be distributed to all participating centers.

Principal investigators will be required to sign the new protocol signature page to confirm their approval of the amendments. The monitoring team and the subsidizing party will be notified. Moreover, the Clinicaltrials.gov registration will be revised in response to any modifications.

#### Consent.

When patients are found eligible for reirradiation by the local multidisciplinary neuro-oncology tumor board, they will be asked by the treating physician if they are willing to be informed about the study. Subsequently, study information will be provided to the patient. Prior to radiotherapy preparations, that will be typically some days later, patients will be asked for consent by the GCP-trained researcher or physician. After informed consent have been obtained, the baseline characteristics will be collected.

#### Ancillary and post-trial care.

Aside from a reduced number of hospital visits for the intervention group, no individual benefits and no known risks are associated with participation in this trial. Although previous studies have suggested that the reirradiation schedule in the intervention group is safe [32,34] there is a theoretically increased risk of radionecrosis. Given the low incidence of radionecrosis in previous cohorts, we think that the benefits (shorter treatment) outweigh this minimal increase in risk. To reduce patients’ burden hospital visits are limited to standard follow-up. Most additional follow-up assessments will be performed by phone call or by self-administered forms. We choose to use the EQ-5D-5L at time points 2, 4 and 6 weeks and thereafter the QLQ-C15-PAL instead of the QLQ-C30 to asses HRQoL. The questionnaires can be completed in less than 10 minutes at home.

Additionally, during a phone call of a few minutes patients will be asked about AEs, anti-edema treatment and reminded to complete their HRQoL assessments.

The sponsor has a liability insurance which is in accordance with article 7 of the WMO. The sponsor also has an insurance which is in accordance with the legal requirements in the Netherlands (Article 7 WMO). This insurance provides cover for damage to research subjects through injury or death caused by the study. The insurance applies to the damage that becomes apparent during the study or within 4 years after the end of the study.

For research-specific visits, i.e., visits that are not part of routine clinical follow-up, travel and parking cost will be reimbursed.

#### Trial status at time of submission.

The study (RISinG trial A) was approved in April 2020. The first participant was recruited on 28-10-2020. The current protocol (RISinG trial B) is version 9, date 04-06-2024. At the time of submission, the trial is ongoing. Recruitment is planned to be completed by November 2026. Data collection, including follow-up of up to one year after the final inclusion, is expected to be completed in Q4 of 2027. Study results are expected to become available shortly after completion of data collection.

## Discussion

The RISinG trial aims to determine whether reirradiation in 4 fractions is non-inferior to reirradiation in 10 fractions, in terms of overall survival after treatment for recurrent glioma.

RISinG trial A was designed as an open-label, randomized, non-inferiority phase III trial with 1:1 allocation for 130 patients, but failed to recruit according to planning. In the RISinG trial A, patient inclusion was challenging due to a combination of factors, including willingness for randomization among potential participants. To address this issue, the inclusion process is simplified in the current trial design (RISinG trial B). RISING trial B will be a phase II, multi-center, clinical trial with a historic control group. To ensure that the analyses are as unbiased as possible, we used modern analytical techniques, including propensity-matched analysis.

In conclusion, the trial in its currently presented form offers the opportunity to evaluate the effects of the different reirradiation schedules for all clinically relevant outcome measures.

### Dissemination policy

The results of the RISinG trial will be disseminated through multiple channels. Findings will be published as open-access scientific papers in peer-reviewed journals and presented at national and international neuro-oncology conferences.

Radiation oncologists participating in the trial, as well as those involved in the Landelijk Platform Radiotherapie Neuro-Oncologie (LPRNO), will be updated through regular meetings.

Patient representatives from the Dutch Neuro-Oncology patient platform (LWNO) or the UMC Utrecht neuro-oncology sounding board group will be involved in the development and communication of study progress and results.

Following publication of main outcomes, we will explore implementation pathways, such as a decision-making support tool.

## Supporting information

S1 File Completed SPIRIT 2025 checklist. (DOCX)

S2 File Structured summary WHO Trial Registration Set. (DOCX)

S3 File Protocol versions, date and changes made. (DOCX)

S4 File Protocol approved by ethics committee, latest version. (PDF)
