# Supplementary material for: Study protocol for a multicenter phase II prospective externally controlled non-inferiority trial of hypofractionated re-irradiation in patients with recurrent high-grade glioma (RISinG)
Source: PLoS One. 2026 Feb 9;21(2):e0342337. doi: 10.1371/journal.pone.0342337 (PMC12885293; doi:10.1371/journal.pone.0342337)
Supplement: S2 File — (DOCX) [file pone.0342337.s002.docx]

## Structured Summary (WHO trial registration dataset)

Primary registry and trial identifying number
Our trial is registered in the Dutch national registry/Netherlands Trial Register (NTR), which is hosted on the CCMO platform. The trial ID in the CCMO register is NL72766.041.20. Registered at 07-04-2020. It was previously registered under the NTR ID: NL-OMON52643.

Secondary Identifying numbers
The project number of the subsidizing party (KWF dutch cancer society) is 12109. The Medical Ethics Review Committee protocol number is 20-056/A.

Source(s) of monetary or material support
Subsidizing party is KWF Dutch Cancer society. Project number 12109.

Primary sponsor
The primary sponsor is UMC Utrecht. UMC Utrecht has the leading role in the design, collection, management, analysis, interpretation of data, writing of the report and the decision to submit. The KWF Dutch Cancer Society does not have a role in the design, execution, analysis, manuscript writing and/or decision to publish.

Contact for public queries
Coordinating investigator: Anouk de Jong
[rtglioma@umcutrecht.nl](mailto:rtglioma@umcutrecht.nl)
+3188-7550813
Department of Radiation Oncology
Division of Imaging and Oncology
University Medical Center Utrecht
Heidelberglaan 100
3508 GA Utrecht
The Netherlands

Contact for scientific queries
Principal Investigator: Joost Verhoeff
[joost.verhoeff@amsterdamumc.nl](mailto:joost.verhoeff@amsterdamumc.nl)
+3120-4441571
Department of Radiation Oncology
Amsterdam University Medical Center
De Boelelaan 1117
1081 HV Amsterdam

Public title
The RISinG trial – optimizing reirradiation for malignant brain tumors

Scientific title
Reirradiation Schedules in Glioma (RISinG): A phase II Prospective Externally Controlled Non-Inferiority Trial

Countries of recruitment
The Netherlands

Health condition(s) or problem(s) studied
Adult patients with a recurrent high-grade glioma

Intervention(s)
Hypofractionated radiotherapy: 4 fractions of 7.5 Gy Simultaneous-Integrated Boost (SIB) in 2 weeks: to the Planning Target Volume (PTV). The historic control group has received 10 fractions (standard-of-care) with a biologically equivalent dose on surrounding brain tissue.

Key inclusion and exclusion criteria
Inclusion criteria include: a supratentorial recurrent grade 3 and 4 glioma with contrast enhancement on contrast-enhanced T1-weighted imaging (CE-T1 MRI), unifocal glioma (i.e. lesions clustering around residual surgical cavity), prior course of treatment including radiotherapy with an EQD2 (α/β = 2) of at least 47 Gy, age ≥ 18 years, Karnofsky Performance Scale ≥ 60, ability of subject to understand nature and individual consequences of the clinical trial, eligible for treatment based on MRI-results (e.g. T1-CE diameter > 6 cm, reflecting a spherical tumor of 125cc).

Exclusion criteria include: previous reirradiation or prior radiosurgery or prior treatment with interstitial radioactive seeds, CE-T1 MRI tumor diameter > 6 cm (reflecting a spherical tumor of 125cc), time interval < 6 months after prior radiotherapy, time interval < 3 weeks after last re-resection (1 week for biopsy), known malignancy < 3 years ago requiring immediate treatment and/or interfering with study therapy and women of childbearing potential without adequate contraception.

Study type
Phase II, multi-center, clinical trial with a historic control group

Date of first enrollment (planned)
The first participant was enrolled at November 16^th^, 2020.

Sample size
The sample size amounts to 165 subjects: 99 in the control group and 66 in the treatment group. At time of submission 33 subjects were included in the experimental arm.

Primary outcome(s)
Outcome name: Overall Survival
Metric/method of measurement: Time from start of reirradiation to death from any cause
Timepoint: Assessed continuously from start of reirradiation, with primary analysis at one year of follow-up

Key secondary outcome(s)
Outcome name: Progression-Free Survival
Metric/method of measurement: time from start of reirradiation until the date of MRI evidence of tumor recurrence or death due to any cause
Timepoint: Assessed continuously from start of reirradiation, with primary analysis at one year of follow-up

Outcome name: Health-Related Quality of Life
Metric/method of measurement: EQ-5D-5L, EORTC QLQ-C15-PAL and EORTC-QLQ-BN20+ 2
Timepoint: 2, 4, 6, 13, 26, 39 and 52 weeks after start of reirradiation

Outcome name: Recurrence patterns
Metric/method of measurement: the proportion of the recurrent lesion located within the prescription 95% isodose surface (D95) of the PTV
Timepoint: assessed at time of radiologic progressions, up to one year of follow-up

Outcome name: Toxicity (specifically clinically relevant radionecrosis),
Metric/method of measurement: incidence and CT-CAE grade of toxicity, including time to toxicity
Timepoint: from start of reirradiation up to one year of follow-up

Outcome name: use of anti-edema treatment (including dexamethasone and bevacizumab)
Metric/method of measurement: recording whether patients receive anti-edema treatment, including details on dose and duration for those treated
Timepoint: from start of reirradiation up to one year of follow-up

Ethics review
Status: approved
Date of approval: march 24^th^, 2020
Name and contact details of Ethics committee: METC NedMec, [metc@nedmec.nl](mailto:metc@nedmec.nl?subject=metc%40nedmec.nl)

Individual trial participating data sharing statement
The pseudonymized individual participant data (IPD) from this trial will be shared with participating hospitals and other neuro-oncological centers involved. Data use is restricted to neuro-oncological research purposes only. The final dataset remains the property of the project.
