# Supplementary material for: Study protocol for a multicenter phase II prospective externally controlled non-inferiority trial of hypofractionated re-irradiation in patients with recurrent high-grade glioma (RISinG)
Source: PLoS One. 2026 Feb 9;21(2):e0342337. doi: 10.1371/journal.pone.0342337 (PMC12885293; doi:10.1371/journal.pone.0342337)
Supplement: S3 File — (DOCX) [file pone.0342337.s003.docx]

| Protocol version | Date | Changes made |
| --- | --- | --- |
| 1 | 03-02-2020 | n.a. |
| 2 | 18-03-2020 | - Inclusion criteria refined to ensure a more homogeneous patient population, based on METC feedback. - Cognitive testing schedule adjusted to start at 6 months follow-up to better align with expected survival, per METC suggestion. - Study dossier reformatted to multicenter format to facilitate participation of additional centers. |
| 3 | 24-03-2021 | - Addition of 4 new participating centers: Maastro, Amsterdam UMC, RadiotherapieGroep and Haaglanden MC - Clarifications in protocol and patient information sheet based on early study phase observations, including dose constraints and organs-at-risk updated in protocol appendix to reflect current clinical practice. |
| 4 | 04-05-2022 | - Addition of 1 new participating center: Verbeeten Instituut Tilburg - Clarifications in protocol and patient information sheet based on early study phase observations, including refinement of GTV (Gross Tumor Volume) and CTV (Clinical Target Volume) definitions and use of SLIM database for data management in multicenter phase |
| 5 | 29-11-2022 | - Addition of 2 new participating centers: Isala Klinieken, Medisch Spectrum Twente - Clarification in protocol regarding adverse event reporting |
| 6 | 30-11-2023 | - Changes in study design: due to low accrual, study design was changed to a signle-arm trial with a historic control group. Consequently, the name was changed to: A Phase 3 Prospective Externally Controlled Non-inferiority Cohort Trial to Compare the Efficacy of Re-Irradiation Schedules in Glioma (RISinG). - Addition of 1 new participating center: UMC Groningen - Change of principle investigators - Change of inclusion database from SLIM to LDot |
| 7 | 04-03-2024 | - Clarification of safety of experimental treatment, adaptations in patient information and clarification of matching procedure |
| 8 | 19-04-2024 | - Sample size recalculation, resulting in updated target patient numbers - Clarification regarding treatment approach for reirradiation in high-grade gliomas |
| 9 | 04-06-2024 | - Changes in protocol: addition of propensity scores to analysis section and inverse probability weighting (IPW) instead of matching |
