# Supplementary material for: Study protocol for a multicenter phase II prospective externally controlled non-inferiority trial of hypofractionated re-irradiation in patients with recurrent high-grade glioma (RISinG)
Source: PLoS One. 2026 Feb 9;21(2):e0342337. doi: 10.1371/journal.pone.0342337 (PMC12885293; doi:10.1371/journal.pone.0342337)
Supplement: S4 File — (PDF) [file pone.0342337.s004.pdf]

A phase II Prospective Externally Controlled Non-Inferiority Cohort Trial to Compare the Efficacy of Re-Irradiation Schedules in Glioma (RISinG)

Re-Irradiation Schedules in Glioma (RISinG) - study

Version 9, 04-06-2024

A phase II Prospective Externally Controlled Non-Inferiority Cohort Trial to Compare the Efficacy of Re-Irradiation Schedules in Glioma (RISinG)

**Protocol ID**	**NL72766.041.20**
**Short title**	**Re-Irradiation Schedules in Glioma (RISinG) - study**
**Version**	**9**
**Date**	**04-06-2024**
**Principal investigator**	***Dr. ir. Marielle E.P. Philippens, MD***<br>Medical physicist radiotherapy, UMCU Utrecht<br>☏+31 (0)88 75 60474<br>🖃M.Philippens@umcutrecht.nl
**Principal Investigator Participating Centers**	***Danielle Eekers, MD PhD***<br>Radiation Oncologist, Maastro<br>☏ +31 (0)88 44 55 600 (algemeen nummer)<br>***Joost J.C. Verhoeff, MD PhD***<br>Radiation Oncologist, Amsterdam UMC<br>🖃 j.j.verhoeff@amsterdamumc.nl<br>***Gerda Wester, MD PhD***<br>Radiation Oncologist, RadiotherapieGroep<br>☏ +31 (0)88 – 790000<br>***Jaap Zindler, MD PhD***<br>***Radiation Oncologist, Haaglanden MC***<br>☏ +31 (0)88 – 979 2013

<u>Tom Rozema, MD PhD</u>

Radiation Oncologist, Verbeeten Instituut

☏ +31 (0)13 594 7769

<u>Hanne Vos, MD PhD</u>

Radiation Oncologist, Isala Klinieken

☏ +31 (0)88 624 5449

<u>Matthijs van der Meulen, MD PhD</u>

Neurologist, Medisch Spectrum Twente

☏ +31 (0)53 487 2850

<u>Miranda Kramer, MD PhD</u>

Radiation Oncologist, UMC Groningen

☏ +31 (0)50 361 2711

**Writing Committee**	**Department of Radiation Oncology, UMC Utrecht**<br>Arthur T.J. van der Boog, MD MSc<br>Cathalijne C.B. Post, MD<br>Joost J.C. Verhoeff, MD PhD<br>**Department of Radiology, UMC Utrecht**<br>Jan Willem Dankbaar, MD PhD<br>Jeroen Hendrikse, MD PhD<br>**Department of Radiation Oncology, UMC Groningen**<br>Mart A.A.M. Heesters, MD PhD<br>Miranda C.A. Kramer, MD PhD<br>**Department of Radiation Oncology, Amsterdam UMC**<br>Frank J. Lagerwaard, MD PhD<br>**Department of Neurology, UMC Utrecht**<br>Tom. J. Snijders, MD PhD<br>**Department of Neurosurgery, UMC Utrecht**<br>Pierre A. Robe, MD PhD<br>**Department of Oncology, UMC Utrecht**<br>Filip Y.F. de Vos, MD PhD
**Sponsor**	University Medical Center Utrecht
**Subsidising party**	KWF
**Independent expert (s)**	J.H.A. Tersteeg, Radiation Oncologist<br>University Medisch Centrum Utrecht<br>☏ +31 (0)88 75 530 39<br>🖃 J.H.A.Tersteeg@umcutrecht.nl

PROTOCOL SIGNATURE SHEET

Name	Signature	Date
**Research Manager:**<br>Prof. dr. D.W.J.Klomp		**04-06-2024**
**Project leader/Principal Investigator:**<br>Dr. Ir. M.E.P. Philippens<br>Clinical physicist radiotherapy department<br>UMC Utrecht		**04-06-2024**

TABLE OF CONTENTS

1. INTRODUCTION AND RATIONALE 12
 1.1 Recurrent glioma and radiotherapy schedules 12
 1.2 Health Related Quality of Life in recurrent glioma 13
 1.3 Adverse Events after brain re-irradiation 13
 1.4 Molecular analysis 14
 1.5 Treatment volume 14
2. OBJECTIVES 16
 2.1 Primary Objective: 16
 2.2 Secondary Objective(s): 16
3. STUDY DESIGN 17
4. STUDY POPULATION 18
 4.1 Population 18
 4.2 Inclusion criteria 18
 4.3 Exclusion criteria 19
 4.4 Sample size calculation 19
5. TREATMENT OF SUBJECTS 21
 5.1 Treatment Protocol 21
 5.2 Experimental treatment arm (arm 1) 21
 5.3 Standard treatment arm (historic controls, arm 2) 21
 5.4 Use of co-intervention 21
6. METHODS 22
 6.1 Study parameters/endpoints 22
  6.1.1 Main study parameter/endpoint 22
  6.1.2 Secondary study parameters/endpoints 22
  6.1.3 Other study parameters 22
 6.2 Study procedures Arm 1 22
 6.3 Study procedures retrospective Arm 2 23
  6.3.1 Health Related Quality of Life (arm 1) 23
  6.3.2 Procedures for Assessing Adverse Events (arm 1 and 2) 24
 6.4 Withdrawal of individual subjects 24
 6.5 Replacement of individual subjects after withdrawal 24
 6.6 Follow-up of subjects withdrawn from treatment 24
 6.7 Premature termination of the study 24
7. SAFETY REPORTING 25
 7.1 Temporary halt for reasons of subject safety 25
 7.2 AEs, SAEs and SUSARs 25
  7.2.1 Adverse events (AEs) 25
  7.2.2 Serious adverse events (SAEs) 25
 7.3 Follow-up of adverse events 26
 7.4 Data Safety Monitoring Board (DSMB) 26
8. STATISTICAL ANALYSIS 27

8.1 Study parameter(s) 27
8.1.1 OS and PFS 27
8.1.2 Recurrence patterns 28
8.1.3 HRQoL in arm 1 28
8.1.4 Toxicity 28
8.1.5 Prognostic and predictive factors 29
8.2 Other study parameters 29
8.2.1 Demographic and baseline characteristics 29
8.3 Interim analysis 29
9. ETHICAL CONSIDERATIONS 31
9.1 Regulation statement 31
9.2 Recruitment and consent 31
9.3 Benefits and risks assessment, group relatedness 31
9.4 Compensation for injury 31
9.5 Incentives 31
10. ADMINISTRATIVE ASPECTS, MONITORING AND PUBLICATION 32
10.1 Handling and storage of data and documents 32
10.1.1 Data Collection 32
10.1.2 Safeguard of key to code 33
10.1.3 Data extraction 33
10.1.4 Data sharing with participating hospitals 34
10.2 Monitoring and Quality Assurance 34
10.3 Amendments 34
10.4 Annual progress report 34
10.5 Temporary halt and (prematurely) end of study report 34
10.6 Public disclosure and publication policy 34
11. REFERENCES 35
Appendix 39
1. Radiotherapy Treatment Protocol for arm 1 39
1.1 Equipment and Planning 39
1.2 Immobilization 39
1.3 Data acquisition and Definitions of Target Volumes and Organs at Risk 39
1.3.1 Gross Tumour Volume (GTV) 39
1.3.2 Clinical Target Volume (CTV) 39
1.3.3 Planning Target Volume (PTV) 39
1.3.4 Organs at Risk (OAR) 39
1.3.5 Recovery potential 42
1.4 Treatment planning 42
1.5 Prescribed dose and fractionation 43
1.6 Dose Limitation to Organs at Risk 43
1.7 Timing of radiotherapy 44
1.8 Treatment Protocol Overview Table 45
2. Toxicity assessed by CTCAE v5.0 46

LIST OF ABBREVIATIONS AND RELEVANT DEFINITIONS

**ABR**	**ABR form, General Assessment and Registration form, is the application form that is required for submission to the accredited Ethics Committee (In Dutch, ABR = Algemene Beoordeling en Registratie)**
**AE**	**Adverse Event**
**AR**	**Adverse Reaction**
**BROK**	**Basic Course for Clinical Investigators; in Dutch: Basiscursus Regelgeving en Organisatie voor Klinisch onderzoekers**
**CA**	**Competent Authority**
**CCMO**	**Central Committee on Research Involving Human Subjects; in Dutch: Centrale Commissie Mensgebonden Onderzoek**
**COWA**	**Controlled Oral Word Association Test**
**CTCAE**	**Common Terminology Criteria for Adverse Events**
**CTV**	**Clinical target volume**
**CV**	**Curriculum Vitae**
**DSMB**	**Data Safety Monitoring Board**
**EU**	**European Union**
**FTP**	**File Transfer Protocol**
**GBM**	**Glioblastoma Multiforme**
**GCP**	**Good Clinical Practice**
**GDPR**	**General Data Protection Regulation; in Dutch: Algemene Verordening Gegevensbescherming (AVG)**
**GTV**	**Gross Target Volume**
**HVLT-R**	**Hopkins Verbal Learning Test-Revised**
**HRQoL**	**Health Related Quality of Life**
**IB**	**Investigator’s Brochure**
**IC**	**Informed Consent**
**ICRU**	**International Commission on Radiation Units and Measurements**
**IDH**	**Isocitrate dehydrogenase gene**
**IMRT**	**Intensity Modulated Radiation therapy**
**KPS**	**Karnofsky Performance Status**
**NCF**	**Neurocognitive function**
**LPRNO**	**Landelijk Platform Radiotherapie Neuro-Oncologie**
**LWNO**	**Landelijke Werkgroep Neuro-Oncologie**
**MGMT**	**Methyl-guanine methyl transferase**

**METC**	**Medical research ethics committee (MREC); in Dutch: medisch ethische toetsing commissie (METC)**
**(m)OS**	**(Median) Overall Survival**
**NVRO**	**Nederlandse Vereniging voor Radiotherapie en Oncologie**
**OAR**	**Organs at Risk**
**PFS**	**Progression Free Survival**
**PTV**	**Planning Target Volume**
**ReRT**	**Re-irradiation**
**(S)AE**	**(Serious) Adverse Event**
**SIB**	**Simultaneous Integrated Boost**
**Sponsor**	**The sponsor is the party that commissions the organisation or performance of the research, for example a pharmaceutical company, academic hospital, scientific organisation or investigator. A party that provides funding for a study but does not commission it is not regarded as the sponsor, but referred to as a subsidising party.**
**SUSAR**	**Suspected Unexpected Serious Adverse Reaction**
**TMT**	**Trail Making Test**
**UAVG**	**Dutch Act on Implementation of the General Data Protection Regulation (in Dutch: Uitvoeringswet AVG)**
**UMC**	**University Medical Center**
**VMAT**	**Volumetric Modulated Arc Therapy**
**WHO**	**World Health Organisation**
**WMO**	**Medical Research Involving Human Subjects Act (in Dutch: Wet Medisch-wetenschappelijk Onderzoek met Mensen**

SUMMARY

Rationale: Re-irradiation is a generally accepted method for salvage treatment in patients with recurrent glioma. However, no standard radiation regimen has been defined. Hypofractionation with dose-escalation will reduce patient’s burden while maintaining the survival benefit.

Objective: To determine if re-irradiation in 4 fractions is non-inferior to 10 fractions in the primary endpoint of survival after re-irradiation. Secondary objectives are to establish and compare recurrence patterns, progression free survival, toxicity and anti-edema treatment and to describe health-related quality of life in the experimental arm.

Study design: RISING trial A was an open label, randomized, non-inferiority, phase III trial with 1:1 allocation for 130 patients among all participating centers. RISING trial B will be a phase II, multi-center, clinical trial with a historic control group.

Study population: Adults with unifocal recurrent high-grade glioma up to 125 cc eligible for re-irradiation.

Intervention: The experimental group receives 4 stereotactic fractions. The historic control group has received 10 fractions (standard of care) with approximately a biological equivalent dose.

Main study endpoint: The primary endpoint is overall survival after re-irradiation.
The key secondary endpoint is progression free survival. Other secondary endpoints are recurrence patterns, toxicity, HRQoLand anti-edema treatment.

Nature and extent of the burden and risks associated with participation, benefit and group relatedness: For the patients included in the study, no individual benefits and no known risks are associated with the tests performed. To reduce patients’ burden, hospital visits are mainly limited to standard follow-up. Most additional follow-up assessments will be performed by telephone or by self-administered forms. We use the EQ-5D-5L at baseline and 2, 4 and 6 weeks after start of radiotherapy and thereafter the QLQ-C15-PAL to assess HRQoL. The questionnaires could be conducted in less than 10 minutes at home. Additionally, at the same time points patients will be asked about adverse events, anti-edema treatment and reminded to complete their HRQoL assessments during a phone call of a few minutes. Furthermore, technical data, including MRI, CT and radiation treatment plans, acquired in standard care will be utilized.

1. INTRODUCTION AND RATIONALE

1.1 Recurrent glioma and radiotherapy schedules

The diagnosis of high grade glioma is usually associated with an extremely poor prognosis, with a median overall survival time (mOS) for glioblastoma (GBM) patients of 14.6 months [1, 2]. Despite multimodality treatments, the local recurrence rate persists to be high (nearly 90% within 2 years) [1, 3]. Additionally, primary WHO grade II gliomas will eventually recur as a high grade glioma at a median of 61 months after primary treatment [4, 5]. Brain tumors have the highest average number of years of life lost of all cancers at 20.1 years, for example compared to prostate cancer at 6.1 years [6].

No standard salvage treatment for this orphan disease has been defined [4, 7, 8]. Next to re-resection and chemotherapy, re-irradiation (ReRT) can be considered as a safe and effective option, leading to mOS ranging from 7.7 to 11.5 months from ReRT start [9–15]. Next to re-resection and chemotherapy, re-irradiation (ReRT) can be considered as a safe and effective option, leading to mOS ranging from 7.7 to 11.5 months from ReRT start. Hypofractionated re-irradiation is shown to be safe and effective in earlier studies: no early or late toxicities above grade 2 (not requiring medical intervention), according to the CT-CAE criteria were observed. Acute toxicity observed in earlier studies included fatigue, headaches, nausea and alopecia and skin erythema. In one study, this was noted in 15% of the patients, but was all < grade 2. Late toxicity includes radionecrosis, this was observed in few patients. In a study by Combs at all, in only 1 out of 233 patients radiation induced necrosis was radiographically diagnosed and histologically confirmed and no other severe early or late side effects above grade 2 could be documented. Pinzi et al observed radiation necrosis in 6% of patients with large volume lesions and this was associated with neurological deterioration. Radionecrosis data reported in the literature indicate an average of 5-14%.

Traditionally, total radiotherapy dose is delivered in several fractions to allow a high enough dose without exceeding normal tissue tolerance. The basis of fractionation is rooted in five primary biological factors: radiosensitivity, repair, repopulation, redistribution and reoxygenation. In conventional schedules the dose is delivered in fractions of approximately 2 Gy to spare normal tissues and maximize the effect of radiation on cancer cells, since the repair capacity of late-reacting normal tissues are better. However, radiotherapy techniques have been improved, allowing more precisely delivered treatment. This provides opportunities for hypofractionation, especially in the case of recurrent glioma, representing patients with a poor prognosis. Hypofractionation is a treatment regimen that delivers higher doses of radiation in fewer fractions, so it provides reduced treatment time and less hospitals visits. In addition, it may achieve increased cell kill from a higher dose per fraction. Nowadays, hypofractionation is frequently used in the treatment of recurrent glioma, appearing to be safe and effective [12, 13, 16–19]. However, there is a lack of information on the effect of different fractionation regimens. Research until now is mostly limited to retrospective single center studies with only one ReRT schedule, as also explained in a comprehensive systematic review on ReRT in glioblastoma [20].

Few studies compared OS in various treatment regimens while taking into account possible confounders. A retrospective cohort study analyzing 121 patients re-irradiated at the UMC Utrecht and UMC Groningen showed no statistically significant difference in OS between patients treated with conventional fractionated radiotherapy (<3Gy/fraction), hypofractionated radiotherapy (3-5Gy/fraction) or stereotactic radiotherapy (≥5Gy/fraction). Also not after correcting for Karnofsky performance status (KPS) (≥< 70%), time interval, planning target volume (PTV) and initial WHO grade [21]. Another single center cohort study compared schedules with 6 fractions of 5 Gy, 12 fractions of 3 Gy and 23 fractions of 2 Gy to a schedule with 18 fractions of 2 Gy in 199 patients. mOS was respectively 9.6, 6.7, 11.3 and 7.6 months. The difference between the groups was significant in univariate (p=0.038) and multivariate analysis (p=0.018) including the covariates initial WHO grade, age (≥<50 years), time interval (>≤12 months), KPS (≥< 80%), neurological symptoms, PTV (≥<47ml) and dose group. This difference was mainly caused by the contrast between the two normofractionated groups (HR 2.45; CI: 1.11-5.38) in favor of the higher biological dose of 46 Gy. The heterogeneous dose definitions, tumor- and patient characteristics among studies makes comparison between studies difficult. For these reasons, still no standard protocol for ReRT of gliomas has been defined yet.

The LPRNO, Landelijk Platform Radiotherapie Neuro Oncologie, conducted an overview of the standard used ReRT schedules among centers in the Netherlands. Ten different standard regimens are used among 13 centers. Moreover, every center uses different regimens next to their standard regimen for the 2 to 6 patients yearly treated. This indicates the large treatment variability. A commonly used schedule is 10 fractions of 3.5 Gy. We aim the best regime for every individual patient. Further research is necessary to achieve this. Participation in current or expected national studies on recurrent gliomas is not hampered by re-irradiation.

1.2 Health Related Quality of Life in recurrent glioma

Thus far, Health Related Quality of Life (HRQoL) measurement (NCF) has been poorly investigated in this setting. Given the overall poor prognosis and high prevalence of impaired functioning in patients with recurrent glioma, HRQoL is of extreme importance to patients and their caregivers [22]. A regimen with few fractions could be assumed to contribute to better preservation of HRQoL. These patient-centered outcomes can provide supportive information about the clinical benefit of the therapy and may facilitate patients’ and physicians’ decision-making.

1.3 Adverse Events after brain re-irradiation

Adverse events (AEs) in ReRT retrospective studies are often poorly registered and therefore underestimated. Most larger retrospective studies do not report severe acute toxicity or radionecrosis at all [9, 10, 14, 15]. Only few studies, including one phase-I prospective study, reported severe acute toxicity [23, 24]. Some studies reported high rates of radionecrosis, including 30% radionecrosis in the SRT group of the retrospective study of

Cho et al. [25], 36% reported late adverse events in the prospective study by Shepherd et al. [26] and 43% in the minority of patients that were progression-free 10 weeks after treatment in the prospective study of Moller et al. [23]. In the pooled results of a meta-analysis on ReRT in glioblastoma, grade ≥3 toxicity rates were poorly reported, but considered to be 4-10%, with higher rates in prospective studies [20]. Real difference in toxicity could be assigned to differences in radiotherapy prescription and patient characteristics. However, the large variation among studies is probably for the most part caused by insufficient detection in retrospective studies. It is of great importance to accurately record and analyze AEs after ReRT to assess safety and its influence on HRQoL.

1.4 Molecular analysis

Molecular markers are of increasing importance in daily practice. Modern neuro-oncology makes use of molecular analysis, so also in this context molecular analysis is necessary. They are a useful addition for diagnosis and treatment guidance due to their predictive and prognostic value. Determination of these markers depends on the diagnostic and therapeutic context.

Isocitrate dehydrogenase gene (IDH) mutations are hallmarks of low-grade glioma. IDH-mutated tumors are associated with a more favorable prognosis. IDH-mutated GBM patients even have a better prognosis than non-mutated grade III astrocytoma patients. Less than 10% of the GBM patients carry a IDH mutation. Approximately 60% of the grade III tumors are IDH-mutated. [4]

Methyl-guanine methyl transferase (MGMT) gene promoter methylation has been correlated with a response to alkylating agent chemotherapy and thus improved outcome in GBM. In low grade IDH-mutated tumors, MGMT methylation is correlated with a better prognosis irrespective of the applied treatment [4].

1.5 Treatment volume

Especially in the case of re-irradiation, toxicity should be taken into account. For this reason, a policy with diameters not exceeding 5cm and small margins is performed in practice. However, since the maximum safe ReRT volume is unknown, this may lead to undertreatment of patients with larger tumors. In the study of Shen et al. with a median ReRT volume of 202cc, volume was not associated with differences in side effects or survival such that larger volume might not be an appropriate exclusion factor for patients who may otherwise be appropriately treated with ReRT [27]. In the current study the maximum accepted GTV for ReRT will be 125cc (about 6cm diameter). This corresponds to the 90th percentile of the patients re-irradiated in the UMC Utrecht and Groningen study [21]. Several studies performed ReRT in gliomas including or beyond this volume as well for hypofractionated [12, 13, 16–19] as normofractionated regimens [9, 25, 28]. A clinical target volume (CTV) margin of 1 cm will be used to treat the potentially microscopic tumor cells in this zone. A lower dose administered to this zone could limit the risk of radionecrosis and

other adverse events, while a simultaneous integrated boost (SIB) to the contrast enhanced area on T1 MRI may kill tumor cells more effectively.

This phase II clinical trial, -- comparing a 4 fractions regimen with a SIB (experimental arm) to a conventional generally accepted schedule with 10 fractions of 3.5 Gy (retrospective arm) -- will establish the efficacy and safety of these dose prescription methods in tumor with a diameter of up to 6cm (~ 125cc).

2. OBJECTIVES

2.1 Primary Objective:

The primary objective is to determine if ReRT in 4 fractions is non-inferior to 10 fractions in the primary endpoint of survival after ReRT. Secondary, superiority will be tested.

2.2 Secondary Objective(s):

1. To compare progression free survival between the two treatment groups.
2. To describe health-related quality of life (HRQoL) in the experimental treatment group
3. To assess neurocognitive functions in a subgroup of the treatment group.
4. To describe toxicity in the experimental treatment group, and compare grade $\geq$3 toxicity in both groups.
5. To establish and compare recurrence patterns on MRI between the two treatment groups.

3. STUDY DESIGN

The original 2019 RISING-trial (RISING trial A) was designed as an open label, randomized, non-inferiority, phase III trial with 1:1 allocation for 130 patients. In this trial 13 patients were included in the experimental arm and 16 patients were included in the control arm. The updated 2023 RISinG trial (RISING trial B) is designed as a phase II, multi-center, clinical trial with a historic control group. The additional recruitment period of this phase will be approximately 30 months with an active follow-up time of 12 months.

The 13 patients of the RISING trial A will be supplemented until a total of 66 patients is reached. These patients fulfilling the inclusion criteria will get the following treatment:

- Hypofractionated radiotherapy with SIB in 2 weeks
  4 fractions of 7.5 Gy SIB to the GTV
  And 4 fractions of 5.5 Gy to the CTV (GTV + 5mm margin).

We will compare the survival of this group with a retrospective control group of patients, subtracted from the retro-RISinG registry study, a multi-center retrospective nation-wide population based cohort study, in which details of patient-, tumor- and treatment-related data of all patients with a recurrence of a primary brain tumor since January 2002 up to December 2022 in the Netherlands will be collected. We will select 99 matched patients in total with the following treatment:

- Moderately hypofractionated radiotherapy in 2-3 weeks
  10 fractions of 3.5 Gy to the CTV (GTV + 5 mm margin)

An overview of the target volume definitions and dose prescriptions is presented in table A3 in section 1.8 of the Appendix.

This control group will be matched to the patients in the experimental arm on an individual level for:

1. Age;
2. Gender;
3. WHO-grade primary tumor;
4. Time interval in between first radiation and re-irradiation;
5. MGMT-status.

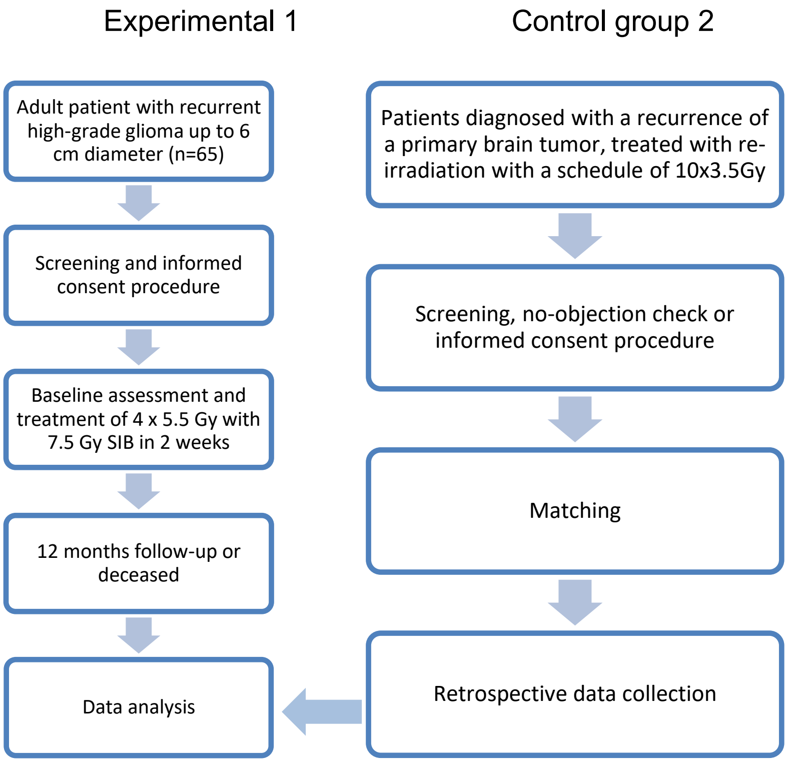

4. STUDY POPULATION

4.1 Population

Patient enrollment in the experimental arm (arm 1) continues until 66 adult patients with recurrent gliomas eligible for re-irradiation are recruited **in total** (RISING A & B) in the participating hospitals. The recruitment period will be approximately 30 months with an active follow-up time of 12 months.
The control group of the original RISinG study (A) had 16 patients. An additional 83 patients will be selected from the retro-RISING study, a multicenter retrospective nation-wide population-based cohort study, in which details of patient-, tumor- and treatment –related data of patients with a recurrence of a primary brain tumor since January 2002 up to December 2022 in the Netherlands will be collected for arm 2. Out of the retro-RISING trial, we will select only the patients with a treatment schedule of 10x3.5Gy. These patients will be individually matched to the patients in the experimental arm.

4.2 Inclusion criteria

In order to be eligible to participate in study arm 1 and 2, a subject must meet all of the following criteria:

- Supratentorial recurrent high-grade glioma with contrast enhancement on CE-T1.
- The gold standard is histological evidence of a recurrence. When a surgical procedure to acquire this evidence is not desirable or possible, a recurrence may be diagnosed by radiological imaging alone, taking the following in consideration:
    - Agreement of the tumor board or a consultant neuro-radiologist that imaging changes are in keeping with recurrence.
    - An interval of no less than 3 months since last (chemo)radiotherapy.

  - Utilization of the RANO criteria for tumor progression [29].
  - If needed, additional imaging sequences such as PET and perfusion MRI.
- Unifocal glioma (i.e. lesions clustering around residual surgical cavity).
- Prior course of treatment including radiotherapy with an EQD2 (α/β = 2) of at least 47Gy.
- Age ≥ 18 years.
- Karnofsky Performance Score 60 or above.
- Ability of subject to understand character and individual consequences of the clinical trial (arm 1).
- Patients who received re-irradiation for the recurrence of a primary brain tumor with a treatment schedule of 10x3.5Gy (arm 2).

4.3 Exclusion criteria

A potential subject who meets any of the following criteria will be excluded from participation in this study (arm 1 and 2):

- Previous re-irradiation or prior radiosurgery or prior treatment with interstitial radioactive seeds.
- CE-T1 tumor diameter greater than 6cm (reflecting a spherical tumor of 125cc).
- Time interval of less than 6 months after prior radiotherapy.
- Time interval of less than 3 weeks after last re-resection (1 week for biopsy).
- Known carcinoma < 3 years ago (excluding Carcinoma in situ of the cervix, basal cell carcinoma, squamous cell carcinoma of the skin) requiring immediate treatment. interfering with study therapy.
- Women with childbearing potential without adequate contraception.

If a patient is not eligible for arm 1 based on the MRI results (e.g. T1-CE diameter >6 cm, reflecting a spherical tumor of 125 cc), these non-eligible patients are being replaced by new patients. These non-eligible patients are not included in the statistical analysis of the trial and treated with the physicians' choice.

4.4 Sample size calculation

The sample size required to achieve a power of 1-β = 0.80 for the one-sided chi-square test at level α = 0.05 when the hazard ratio is actually 0.90, and a distribution of 40% in the intervention group and 60% and the control group, amounts to 165 subjects: 99 in the control group and 66 in the treatment group [30]. The non-inferiority ratio is 1.4. Unfortunately, there are no historical randomized placebo-controlled trials for arm 2 to use to formulate a margin, therefore we collect this data now as well for matched patients. This ratio is based on clinical judgment of the calculated survival fractions at the proposed non-inferiority margins and published survival data [21].
To clarify the meaning of this non-inferiority margin in practice, the non-inferiority margin specific survival fraction at median survival time could be calculated. This non-inferiority margin means in practice that at least 76% of the patients surviving at the median survival

time of the control group would have survived when treated with the experimental treatment. In other words, at least 38 percent of the patients in the intervention group reach the median survival time instead of 50 percent in the control group.
Expected event rate at the minimal follow-up time of 12 months is 0.7 based on the literature [21, 31]. However, a part of the study population may have a longer follow-up period of even up to 3.5 years. It is anticipated that the proportion of subjects observed with the event during the total study period is 0.8. These results assume that the hazard ratio is constant throughout the study and that Cox proportional hazards regression or the non-inferiority log rank test is used to analyze the data. To compensate for any drop-outs or slight deviation from the assumptions made, the total sample size should be 130 patients (10% added to the calculated sample size).
A point of discussion is that we anticipated that the hazard ratio is actually 0.90, however we can in fact not know the actual hazard ratio. It is justified to assume a hazard ratio below 1.0 when hypothesizing that the tumor will be treated slightly better by a higher dose per fraction.

Table 1. Number of patients to include to achieve a power of 0.80 with a non-inferiority ratio of 1.4 at a decease rate of 0.80 for different alfa-levels and actual hazard ratios.

**Alfa/HR**	**0.8**	**0.9**	**1.0**
**0.05**	99	159	274
**0.10**	72	116	200

The trial is primarily designed to show non-inferiority, in addition superiority will be tested. If the actual hazard ratio is lower, the patient number will be sufficient to detect superiority (Table 2).

Table 2. Number of patients to include to achieve a power of 0.80 at a decease rate of 0.80 for different alfa-levels and actual hazard ratios for superiority analysis.

**Alfa/HR**	**0.4**	**0.5**	**0.6**	**0.7**	**0.8**
**0.025**	47	82	151	308	787
**0.05**	37	65	119	243	621
**0.10**	27	47	87	178	453

5. TREATMENT OF SUBJECTS

5.1 Treatment Protocol

Radiotherapy will be planned and delivered in every center according to the treatment protocol (Appendix 1).

5.2 Experimental treatment arm (arm 1)

Since prospective phase III research on the optimal fractionation regimen is sparse, a radiobiological theoretical optimal regimen is chosen from retrospective studies and phase I/II studies. The linear-quadratic model describes cell killing, both for tumor control and for normal tissue complications following exposure to a varying amount of radiation. The most modern method of comparing fractionation schedules is to use the linear quadratic equation to calculated the equivalent dose in 2 Gy fractions (EQD2). For PTV_{boost}, the EQD2 may be up to 40 Gy based on the maximal cumulative dosis of 100 Gy advised by Sminia and Mayer [32, 33]. For this reason, the dose on PTV was selected to be 4 fractions of 5.5 Gy ($EQD2_{\alpha/\beta=2}$ = 41 Gy).

For PTV, the maximal dose should not exceed 8 fractions of 5 Gy ($EQD2_{\alpha/\beta=2}$ = 70 Gy). In a prospective phase I trial, hypofractionated stereotactic radiotherapy was given using 5 Gy per fraction to doses ranging from 20 to 50 Gy on a dose escalation program, in patients with recurrent glioma with a median target volume of 24cc. Exceeding 8 fractions of 5 Gy resulted in 6.4 times more radiation damage [26]. Four fractions of 7.5 were considered safe with a comparable $EQD2_{\alpha/\beta=2}$ of 71 Gy.

Experimental treatment schedule will be 4 fractions of 7.5 Gy SIB to the CE on CE-T1 and 4 fractions of 5.5 Gy to the CE with a 10mm margin.

5.3 Standard treatment arm (historic controls, arm 2)

In 1999, a phase I dose escalation of hypofractionated stereotactic radiotherapy in recurrent or persistent malignant gliomas was conducted. Three different total dose levels were sequentially evaluated: 24.0 Gy in 3.0 Gy fractions (five lesions), 30.0 Gy in 3.0 Gy fractions (10 lesions), and 35.0 Gy in 3.5 Gy fractions (nine lesions). No grade 3 toxicities were observed and the response rate was 78% [34]. From that time, 10 fractions of 3.5 Gy is a generally accepted regimen. Until now, this schedule has shown to be safe and effective in practice [35]. $EQD2_{\alpha/\beta=2}$ of this schedule is 48 Gy.

5.4 Use of co-intervention

Concomitant dexamethasone and anticonvulsant therapy is allowed. Salvage surgery prior to start radiation is allowed. Systemic therapy including chemotherapy, immunotherapy or targeted therapy during ReRT is not allowed.

6. METHODS

6.1 Study parameters/endpoints

6.1.1 Main study parameter/endpoint

The primary endpoint will be the time to death (overall survival (OS)) counted from the first date of ReRT until death due to any cause. Both treatments are finished within 2 to 3 weeks.

6.1.2 Secondary study parameters/endpoints

The key secondary endpoint is:

- Progression free survival (PFS) counted from the first date of re-irradiation until the date of MRI evidence of tumor recurrence or death due to any cause. Agreement of the tumor board or a consultant neuro-radiologist that imaging changes are in keeping with recurrence and not radionecrosis is necessary.

Other secondary endpoints include the following:

- Recurrence Patterns on MRI.
- Radiation toxicity graded by CTCAE v5.0
- Anti-edema therapy (including Dexamethasone and Bevacizumab use).
- Health Related Quality of Life (HRQoL) prospectively monitored by EQ-5D-5L, EORTC QLQ-C15-PAL and EORTC-QLQ-BN20+2 questionnaires. (only for the experimental arm 1, cannot be analyzed retrospectively in the control arm 2).

6.1.3 Other study parameters

In addition, KPS will be recorded in the experimental arm 1 and molecular analysis on prior surgically acquired tumor tissue, as well as at the time of recurrence (if available), will be performed.

6.2 Study procedures Arm 1

After obtaining informed consent (for details, see section 9.2), patients will receive treatment and necessary follow-up procedures. During the course of the study, participating centers will collect baseline characteristics, technical data such as MRI's, CT's and treatment plans and date of death. Medical imaging will be uploaded to a radiological database at a FTP server. Central data collection exists of HRQoL assessment, toxicity, anti-edema therapy and performance status. Central data collection will be performed by the study team by phone call, or via digital or paper questionnaires. Study procedures are summarized in Table 3. Patients will complete HRQoL questionnaires and the study team will record toxicity and anti-edema treatment by phone call at baseline and at 2, 4, 6, 13, 26, 39, and 52 weeks from the start of ReRT or until disease progression (whichever is sooner). During the first 6 weeks HRQoL will be assessed using the EQ-5D-5L. At subsequent time points QLQ-C15-PAL and BN20+2 will be completed digitally or on paper. Clinicians will be asked to record toxicity, KPS and anti-edema therapy use during regularly scheduled consults.
In case of suspicion of progression, recurrence or radionecrosis on MRI without consensus of diagnosis by the tumor board a FDG-PET, FET-PET or spectroscopy may be used to differentiate but is not mandatory.

IDH-1/2 mutation and MGMT-methylation will be determined at baseline using prior surgically acquired tissue, when molecular analysis has not been performed before, as well as at the time of recurrence (if available).
Any clinical follow-up data after 12 months will be uploaded at time of death, if known.

Table 3. Treatment and follow-up procedures per patient in experimental arm 1

	Baseline	Treatment and follow-up period (weeks after first fraction)						
		2	4	6	13	26	39	52
Baseline characteristics and intake	X							
Hypofractionated RT		X						
HRQoL	X	X	X	X	X	X	X	X
Molecular analysis	X							
Upload information/imaging	X					X		X

6.3 Study procedures retrospective Arm 2

The available data for the retrospective control group (arm 2) will be selected from the retro-RISinG trial.

6.3.1 Health Related Quality of Life (arm 1)

Health Related Quality of Life (HRQoL) will be assessed via 3 questionnaires: EQ-5D-5L, EORTC QLQ-C15 PAL and EORTC-QLQ-BN20+2.

EQ-5D-5L is a validated measure of health status developed by the EuroQol Group in order to provide a simple, generic measure of health for clinical and economic appraisal [36, 37]. It consists of a descriptive system comprising 5 dimensions (mobility, self-care, usual activities, pain/discomfort, anxiety/depression) at 5 levels and the EQ Visual Analogue scale. This tool is used for the HRQoL measurement during the first 6 weeks after therapy start, since it takes only a few minutes to complete.
Subsequently, HRQoL will be determined using the EORTC QLQ-C15 PAL questionnaire [38] and QLQ-BN20+2 questionnaire [39] every 3 months after finishing radiotherapy treatment. The QLQ-C15 PAL is a validated general core QoL assessment tool which is developed for (near) palliative cancer care patients. The tool consists of 15 questions: 2 multi-item functional scales (physical and emotional functioning), 2 multi-item symptom scales (fatigue and pain) along with 5 single-item symptom scales (nausea/vomiting, dyspnea, insomnia, appetite loss, and constipation), and 1 final question referring to overall QoL. Patients rate each question/item on a numeric scale from 1 (not at all) to 4 (very much), with the exception of overall QoL, which was rated from 1 (very poor) to 7 (excellent).
The QLQ-BN20+2 includes the QLQ-BN20 questionnaire that has been validated for use in patients with primary brain malignancies. In addition, two scales for concentration and memory were added which were omitted in the abbreviated QLQ-C15-PAL. The QLQ-BN20 consists of 20 questions: 7 single-item symptom scales (headaches, seizures, drowsiness,

hair loss, itchy skin, leg weakness, and bladder control), along with 4 multi-item scales (future uncertainty, visual disorder, motor dysfunction, and communication deficit). The QLQ-BN20+2 was first described by Caissie et al. and validated by Nguyen et al. in patients with brain metastases [39, 40].
These questionnaires consist in total of 37 assessments instead of 50 assessments when using the QLQ-C30. This aims to minimize patients' burden and increase the chance of maintaining follow-up as patients progress through their disease course [39].

6.3.2 Procedures for Assessing Adverse Events (arm 1 and 2)

Early and late AEs associated with radiotherapy will be recorded or retrieved. Data includes (but is not limited to) central nervous system necrosis, edema, seizure, headache, fatigue, nausea and radiation dermatitis. Toxicity will be assessed according to CTCAE version 5.0. See Appendix 2 for toxicity grade per term.

6.4 Withdrawal of individual subjects

Subjects can leave the study arm 1 at any time for any reason if they wish to do so without any consequences. The investigator can decide to withdraw a subject from the study for urgent medical reasons. If a patient is going off protocol treatment, the reason should be documented. Patients are not obligated to specify the reason of withdrawal.

6.5 Replacement of individual subjects after withdrawal

In section 4.4 the sample size calculation for this study is given. Therefore, an inclusion of 65 subjects per study group is aimed to generate a total of 130 patients. If a subject in arm 1 discontinues from the study prematurely the reason, if given, must be fully evaluated and recorded appropriately.
If a patient who has signed informed consent withdraws for any reason before randomization, the patient will be replaced. These patients are not included in the statistical analysis. After randomization, patients withdrawing because of any reason will not be substituted by additional patients. Patients withdrawn from the trial retain their identification codes (e.g. randomization number), if already given. New patients must always be allotted a new identification code.

6.6 Follow-up of subjects withdrawn from treatment

Patients withdrawn from treatment will be asked if they will continue completing the questionnaires and if their medical record may be checked for reported toxicity and progression. Patients will be asked for oral and written informed consent.

6.7 Premature termination of the study

A predefined interim analysis will be performed based on tumor volume as described in section 8.3. No premature termination of the study is expected due to safety issues, because the regimens have been used in practice before with limited toxicity and radionecrosis.

7. SAFETY REPORTING

7.1 Temporary halt for reasons of subject safety

In accordance to section 10, subsection 4, of the WMO, the sponsor will suspend the study if there is sufficient ground that continuation of the study will jeopardize subject health or safety. The sponsor will notify the accredited METC without undue delay of a temporary halt including the reason for such an action. The study will be suspended pending a further positive decision by the accredited METC. The investigator will take care that all subjects are kept informed.

7.2 AEs, SAEs and SUSARs

7.2.1 Adverse events (AEs)

Adverse events are defined as any undesirable experience occurring to a subject during the study, whether or not considered related to re-irradiation treatment. All adverse events reported spontaneously by the subject or observed by the investigator or his staff will be recorded. Since any consequences of the radiotherapeutic treatment are expected to occur shortly after the radiation, AEs will only be reported until 30 days after the last fraction. Only AE's of grade 3 or higher will be reported, as treatment discomfort is inherent to this population. In addition, toxicity will already be registered as part of the study. Adverse events and their grading in glioma patients undergoing radiotherapy are summarized in Appendix 2.

7.2.2 Serious adverse events (SAEs)

A serious adverse event is any untoward medical occurrence or effect that

- results in death.
- is life threatening (at the time of the event).
- requires hospitalization or prolongation of existing inpatients' hospitalization.
- results in persistent or significant disability or incapacity.
- is a congenital anomaly or birth defect.
- any other important medical event that did not result in any of the outcomes listed above due to medical or surgical intervention but could have been based upon appropriate judgment by the investigator.

An elective hospital admission will not be considered as a serious adverse event.

The investigator will report all SAEs to the sponsor without undue delay after obtaining knowledge of the events. However, patients with recurrent glioma have a poor life expectancy. Therefore, a significant proportion of patients will die within the study duration of 1 year after radiotherapy. The SAE sensitive period will be up until 30 days after last ReRT fraction to account for treatment related effects. Expected SAEs are hospitalization,

persistent or significant disability or incapacity, or death due to glioma progression. These expected SAEs will be reported every year in a line listing.

The sponsor will report the SAEs through the web portal *ToetsingOnline* to the accredited METC that approved the protocol, within 7 days of first knowledge for SAEs that result in death or are life threatening followed by a period of maximum of 8 days to complete the initial preliminary report. All other SAEs will be reported within a period of maximum 15 days after the sponsor has first knowledge of the serious adverse events.

7.3 Follow-up of adverse events

All AEs will be followed until they have abated, or until a stable situation has been reached. Depending on the event, follow up may require additional tests or medical procedures as indicated, and/or referral to the general physician or a medical specialist.

7.4 Data Safety Monitoring Board (DSMB)

A DSMB will not be established, due to the low risk of adverse events.

8. STATISTICAL ANALYSIS

8.1 Study parameter(s)

Planned statistical methods for the trial are presented in this chapter. Minor revisions to this statistical analysis plan are allowed. Final statistical analysis plan should be written prior to any formal analysis of the data and finalized prior to end of patient recruitment.

8.1.1 OS and PFS

All statistical tests for the efficacy endpoint OS and PFS will be performed according to intention-to-treat (ITT) principle, which is the most unbiased method. Considering the trial being a non-inferiority trial, which is more sensitive to bias, a secondary per-protocol (PP) analysis will be done for OS [43]. The PP analyses will be performed on a "subset of patients who complied sufficiently with the protocol, such as exposure to treatment, availability of measures and absence of major protocol violations" according to the ICH E9 guidelines. The ITT analysis includes all participants. Start date of ReRT will be used as time is 0. Patients not known to have died during the study will be censored for OS at the day they were last known to be alive. Patients free from progression and alive at the last visit will be censored for PFS at the day of last assessment. Endpoints are measured from the first date of re-irradiation.

Distributions of OS and PFS will be estimated by the Kaplan-Meier method. The OS rates at one year, PFS rates at six months and medians of OS and PFS will be presented with two-sided 95% confidence interval(CI) computed using the log-log transformation.

To minimize the effect of confounding when estimating the difference between the treatment and retrospective control group, stabilized inverse probability of treatment weighting (SIPTW) using propensity scores will be applied when comparing the groups on OS and PFS. Propensity scores will be estimated using logistic regression including important covariates for OS and PFS: primary histology, GTV, KPS and age.

8.1.1.1. *OS*

The hazard rate for the experimental versus the retrospective control group and its two-sided 80 and 95% CIs will be estimated using a Cox proportional hazards model. We will adjust for confounding using propensity scores.. Omission of 4 fractions as non-inferior to 10 fractions of radiotherapy if the higher 90% CI of the hazard ratio (HR) of 4 fractions versus 10 fractions was not above 1.4.

Only when non-inferiority has been demonstrated, we will test for superiority of the experimental treatment in a hierarchical fashion, using Kaplan-Meier method and two-sided log-rank test at a significance level $\alpha = 10\%$ in ITT analysis.

8.1.1.2. *PFS*

The primary comparison of the time-to-event distributions between the two treatment arms will be done using the two-sided log-rank test at significance level $\alpha = 5\%$ The hazard rates for the experimental versus the retrospective control group and its two-sided 95% CI will be

estimated using a Cox proportional hazards model. We will adjust for confounding using propensity scores.

8.1.2 Recurrence patterns

Recurrence patterns will be compared between the arms using logistic regression, also adjusted for confounding using propensity scores.Recurrences are defined as “central” if more than 95% of the tumor recurrence resides within the prescription 95% isodose surface (D95) of the PTV; “in-field” if more than 80% of recurrent lesion is inside D95 of the PTV, and “marginal” if 20% to 80% of the lesion is inside D95 of the PTV. In all other cases, recurrences are defined as outside the radiation field (“ex-field”) according to the study of Lee et al. [44].

8.1.3 HRQoL in arm 1

HRQoL will only be assessed in the experimental group. Reasons for missing baseline and follow-up questionnaires will be assessed. Linear Mixed Effects models will be used for regression analyses of HRQoL. In addition, the repeated measures of the EQ-5D-5L, QLQ-C15-PAL and BN20+2 functional and symptom scales will be assessed by time to deterioration (TTD) analyses. The time until definitive HRQOL score deterioration (TUDD) is defined as time from inclusion to a first deterioration of the score with a minimal clinically important difference (MCID) of at least 10 points as compared to the baseline score. With no further improvement of more than the MCID as compared to the baseline score or if the patient dropped out after deterioration, resulting in missing data. According to the construction of the HRQOL scores, deterioration corresponds to an increase (e.g., for symptomatic scales of the EORTC questionnaires and EQ-5D-5L) or a decrease (e.g., for functional scales) of the score.

Patients with no score available are excluded from the time-to-deterioration analyses. Patients with no baseline score are usually censored at baseline and those with no follow up scores but with a baseline score are censored one day after baseline. Patients with no deterioration before drop-out from the study, including death, are censored at the time of last HRQoL questionnaire completion or last follow-up.

The TUDD estimation will be calculated using the Kaplan-Meier method and described using median and 95% CI.

8.1.4 Toxicity

Toxicity will only be assessed in the experimental group. Analyses of radiotherapy toxicity will be performed in all patients who received at least one fraction. Acute events occurring from start of radiotherapy until 90 days and late events occurring from 91 days after end of radiotherapy will be presented. Further analyses of treatment toxicity will present the grade 3 to 5 acute or late side effect by treatment arm. The time to occurrence of any severe late side effects and separately radionecrosis (distinguishing symptomatic and asymptomatic) will be estimated by cumulative incidence. The time to severe late side effects will be calculated from the time of start of radiation treatment to the first evidence of any grade 3-5 late side effects. Patients alive without grade 3-5 late toxicity will be censored at the date of last

follow-up, patients who died without experiencing late grade 3-5 side effects will be assessed as competing risk at the time of death.

8.1.5 Prognostic and predictive factors

Additional exploratory analyses using Cox regression analyses will study the prognostic impact of factors (other than treatment, age, gender, WHO-grade primary tumor, time interval in between first radiation and re-irradiation and MGMT-status), including baseline KPS, number of recurrence, tumor volume and dexamethasone use at baseline. In addition, the predictive value for the treatment effect of the 4 and 10 fraction regimens of these factors will be investigated using Cox Regression analyses.

8.2 Other study parameters

8.2.1 Demographic and baseline characteristics

Data on demographic and baseline characteristics will be summarized using descriptive statistics. For continuous variables, in case of normal distribution by means and standard deviations, and in case of non-normal distribution by medians and interquartile ranges. For discrete variables data will be summarized by frequencies and proportions.

8.3 Interim analysis

Six months after inclusion of every 10 patients an interim analysis will be performed to review the toxicity data. If the toxicity is unexpectedly high in large tumors (65-125cc), the maximal tumor diameter will be modified to 5 cm (reflecting a spherical tumor of 65 cc).The detailed interim analyses are not made available to trial participants.
Sequential boundaries will be used to monitor dose-limiting toxicity rate. Full follow-up for acute toxicity is defined as 90 days after last irradiation fraction. Full follow-up for radionecrosis is defined as after the MRI at 6 months. This is a Pocock-type stopping boundary that yields the probability of crossing the boundary at most 0.05 when the rate of dose-limiting toxicity is equal to the acceptable rate 30, 20 or 10% depending on adverse event grading [45].

The research team will be responsible for the interim analysis. The accrual of patients with a tumor with a diameter >5 cm will be halted if excessive numbers of dose-limiting toxicities are seen, that is, if the number of dose-limiting toxicities is equal to or exceeds b_n out of n patients with full follow-up (Table 4.1, 4.2 and 4.3). No more interim analyses will be performed if accrual of patients with a tumor volume of >5 cm (reflecting a spherical tumor of 65 cc) is halted based on the above, if after 4 iterations of interim analyses, no excessive numbers of dose-limiting toxicities are seen, as mentioned above or when inclusion is completed without excessive dose-limiting toxicities. In any of these cases, the METC that gave a favorable opinion will be notified.

Table 4.1 The maximal tumor volume will be modified if the number of dose limiting grade 3 to 5 toxicities is equal to or exceeds b_n out of n patients with completed follow-up. This boundary is equivalent to testing the null hypothesis, after each patient, that the event rate is equal to 0.3, using a one-sided level 0.017041 test.

Number of Patients, n	1	2	3	4	5	6	7	8	9	10	11	12	13	14	15	16	17	18	19	20
Gr 3-5 Boundary, b_n	-	-	-	4	5	5	6	6	7	7	8	8	9	9	10	10	10	11	11	12
Number of Patients, n	21	22	23	24	25	26	27	28	29	30	31	32	33	34	35	36	37	38	39	40
Gr 3-5 Boundary, b_n	12	13	13	13	14	14	15	15	15	16	16	16	17	17	18	18	18	19	19	20

Table 4.2 The maximal tumor volume will be modified if the number of dose limiting grade 4 or 5 toxicities is equal to or exceeds b_n out of n patients with completed follow-up. This boundary is equivalent to testing the null hypothesis, after each patient, that the event rate is equal to 0.2, using a one-sided level 0.015124 test.

Number of Patients, n	1	2	3	4	5	6	7	8	9	10	11	12	13	14	15	16	17	18	19	20
Gr 4-5 Boundary, b_n	-	-	3	4	4	5	5	5	6	6	6	7	7	7	8	8	8	9	9	9
Number of Patients, n	21	22	23	24	25	26	27	28	29	30	31	32	33	34	35	36	37	38	39	40
Gr 4-5 Boundary, b_n	9	10	10	10	11	11	11	11	12	12	12	13	13	13	13	14	14	14	15	15

Table 4.3 The maximal tumor volume will be modified if the number of dose limiting grade 5 toxicities is equal to or exceeds b_n out of n patients with completed follow-up. This boundary is equivalent to testing the null hypothesis, after each patient, that the event rate is equal to 0.1, using a one-sided level 0.015673 test.

Number of Patients, n	1	2	3	4	5	6	7	8	9	10	11	12	13	14	15	16	17	18	19	20
Gr 5 Boundary, b_n	-	2	3	3	3	4	4	4	4	4	5	5	5	5	5	6	6	6	6	6
Number of Patients, n	21	22	23	24	25	26	27	28	29	30	31	32	33	34	35	36	37	38	39	40
Gr 5 Boundary, b_n	6	7	7	7	7	7	7	8	8	8	8	8	8	9	9	9	9	9	9	9

The most objective toxicity terms will be evaluated, including central nervous system necrosis, edema and seizures. Maximal accepted percentage grade 3 to 5 radiotherapy related acute toxicity or late radionecrosis (sever symptoms; medical intervention indicated) is 30%. Maximal accepted percentage grade 4 or 5 adverse events is 20%. Maximal accepted percentage grade 5 adverse events is 10%. Toxicity terms are graded according to the CTCAE v5.0 (Appendix 1.0). Edema will be graded similar to radionecrosis.

If maximal tumor diameter has to be modified to 5 cm, expected loss of recruitment amounts 15% based on the retrospective dataset of UMC Utrecht and UMC Groningen. The recruitment time should be prolonged with approximately 5 months to achieve a total of 65 patients.

9. ETHICAL CONSIDERATIONS

9.1 Regulation statement

The trial will be conducted according to the Medical Research Involving Human Subjects Act (WMO) and adheres to the ethical principles that have their origin in the declaration of Helsinki.
Patient privacy is ensured by de-identifying all submitted data and using a subject identification code. All patients will have the right to withdraw from the study at any time during the trial.

9.2 Recruitment and consent

When patients are found eligible for re-irradiation by a multidisciplinary neuro-oncology tumor board, they will be asked by the treating physician if they are willing to be informed on the study. Subsequently, study information will be provided to the patient. Prior to radiotherapy preparations, that will be typically some days later, patients will be asked for consent by the BROK-certified researcher or physician and if the patient is willing to participate, after the inform consent procedure the baseline characteristics will be collected.

9.3 Benefits and risks assessment, group relatedness

Aside from a reduced number of hospital visits for the intervention group, no individual benefits and no known risks are associated with participation in this trial. The radiation doses that will be administered have already proven to be safe in previous studies[26, 35].To reduce patients' burden hospital visits are limited to standard follow-up. Most additional follow-up assessments will be performed by phone call or by self-administered forms. We choose to use the EQ-5D-5L at time points 2, 4 and 6 weeks and thereafter the QLQ-C15-PAL instead of the QLQ-C30 to asses HRQoL. The questionnaires can be completed in less than 10 minutes at home. Additionally, during a phone call of a few minutes patients will be asked about AEs, anti-edema treatment and reminded to complete their HRQoL assessments.

9.4 Compensation for injury

The sponsor has a liability insurance which is in accordance with article 7 of the WMO.

The sponsor also has an insurance which is in accordance with the legal requirements in the Netherlands (Article 7 WMO). This insurance provides cover for damage to research subjects through injury or death caused by the study.
The insurance applies to the damage that becomes apparent during the study or within 4 years after the end of the study.

9.5 Incentives

When the visits are not included in the standard follow-up, travel and parking cost will be reimbursed.

10. ADMINISTRATIVE ASPECTS, MONITORING AND PUBLICATION

10.1 Handling and storage of data and documents

The handling of personal data will comply with the General Data Protection Regulation (GDPR) (In Dutch: Algemene Verordening Gegevensbescherming (AVG)), so data will be handled confidentially and coded.

10.1.1 Data Collection

Study sites will register a patient in the web-based application Ldot after informed consent of the patient. Each user will get a personal account to allow access to his/her own studies, site(s), subjects and specific viewing rights. After registration of the patient, Ldot will generate a unique study registration (USR) number. Access to Ldot can be granted by different roles. A “role based” access to the system will avoid unauthorized data access and prevents users to perform action they are not allowed to do.
Users have access to the system using their personal username and password plus 2FA/SRAM (SURF Research Access Management). The system uses an electronic audit trail to log changes of important variables with timestamps and user information. Ldot data traffic over the internet is encrypted using secured data communication protocols.
Databases and web servers of Ldot application are hosted in data centers that meets the highest available standards for security. Access to the ICTS server rooms is limited by physical and automatic access security, and is supported by fire protection and continuity guarantees in the field of energy supply. The ICTS servers are protected by firewalls and measures have been taken to support random irregularities on the network. The servers are placed in a specially secured VLAN. Data guarantee has been established for each ICT system: the data is regularly backed up and regularly tested to see whether this backup can be restored.
The observational data will prospectively be collected from medical charts, pathology reports, radiology reports, neurocognitive function tests and web-based surveys (PROFILES web-based database). The interfaces will be realized using common, standard and secure software.
Data collected through web-based surveys will be sent to the central RISinG database coded without patient identifiers. Enrolled participants will be assigned study identification codes that will not resemble medical record number, governmental identification or other unique identifiers. Participants who are not able to use the web-based surveys will be offered hardcopy questionnaires instead. Hardcopy questionnaires will be entered in the PROFILES web-based database after secured login. The hardcopy questionnaires will then be physically stored in a secure archive of the UMC Utrecht.

In addition to the pseudonymized clinical data, technical data on imaging and radiation plans will be collected as well. These will be stored in the Research Imaging Architecture (RIA), a

fully secure data storage infrastructure. This storage infrastructure runs tools to manage incoming and outgoing data, verifies the origin (approval) of incoming data requests and several other logistics. These represent a dynamic adaptable toolset (which exists on both entities) that can be used to perform various activities on technical patient data before it is sent or just as it is being received. Example activities include: de-identifying, pseudonymizing and/or anonymizing data, packaging/deflating incoming data using encryption key, verifying (point-to-point) communication, handling incoming duplicated data according to rule set and retrying logic (robustness) in case of network issues or maintenance.

All data will be analyzed by the investigators of this study and the data will be kept for at least 15 years.

10.1.2 Safeguard of key to code

A separate identification log only containing codes with corresponding patient identifiers (name, date of birth, sex and date of inclusion), will be stored on a different secured location within the UMCU to prevent the possibility to directly match study data with patient identifiers. The same manner of coding will be used for all studies within the RISinG project. The participant’s name and personal data will remain confidential and will not be published in any way, however, the sponsor’s monitor or representative and regulatory representatives (FDA and/or European Communities EU Notified Body Representatives), auditors and inspectors may have access to medical files in order to verify authenticity of data collected.

In order to send questionnaires, patient identifiers (name, date of birth, sex and address) necessary to send questionnaires will be stored in the PROFILES web-based database which is accessible after secured login. Employees of PROFILES could have access to these patient identifiers as well. PROFILES is part of the Dutch Cancer Registry (in Dutch: Integraal Kankercentrum Nederland, IKNL) and complies with the General Data Protection Regulation, so data will be handled confidentially and coded. Upon informed consent patients are aware that their patient identifiers will be used by employees of PROFILES in order to send questionnaires.

10.1.3 Data extraction

Requested data can be extracted from the RISinG database by exporting data to Excel, SPSS and/or R. Data will only be exported coded (i.e. without any identifying information such as patient name, initials, social security number).

10.1.4 Data sharing with participating hospitals

Upon informed consent patients are aware that their pseudonymized data may be shared with participating hospitals in favor of conducting the research described in this application. These data will remain property of this project and will only be provided upon request and after quality check at/by the research board of this project.

10.2 Monitoring and Quality Assurance

The monitoring of the study will be performed by a qualified and trained monitor to assure the quality and validity of the research data. The monitoring will be performed according to the NFU (Nederlandse Federatie van Universitaire Medische Centra) guidelines. The monitoring plan is included in the application.

10.3 Amendments

Amendments are changes made to the research after a favorable opinion by the accredited METC has been given. All amendments will be notified to the METC that gave a favorable opinion. Non-substantial amendments will not be notified to the accredited METC and the competent authority, but will be recorded and filed by the sponsor.

10.4 Annual progress report

The sponsor will submit a summary of the progress of the trial to the accredited METC once a year. Information will be provided on the date of inclusion of the first subject, numbers of subjects included and numbers of subjects that have completed the trial, serious adverse events/ serious adverse reactions, other problems, and amendments.

10.5 Temporary halt and (prematurely) end of study report

The investigator/sponsor will notify the accredited METC of the end of the study within a period of 8 weeks. The end of the study is defined as the last patient's last visit.

The sponsor will notify the METC immediately of a temporary halt of the study, including the reason of such an action.

In case the study is ended prematurely, the sponsor will notify the accredited METC within 15 days, including the reasons for the premature termination.

Within one year after the end of the study, the investigator/sponsor will submit a final study report with the results of the study, including any publications/abstracts of the study, to the accredited METC.

10.6 Public disclosure and publication policy

Results will be published with open access unreservedly regardless of their nature in accordance with the CCMO statement on publication policy. The current PhD-student will be first author and the PI last author. Every center that includes patients, added to this protocol in amendments, will be provided authorship.

11. REFERENCES

1. Stupp R, Mason WP, van den Bent MJ, Weller M, Fisher B, Taphoorn MJB, et al. Radiotherapy plus Concomitant and Adjuvant Temozolomide for Glioblastoma. N Engl J Med. 2005;352.
2. Stupp R, Hegi ME, Mason WP, van den Bent MJ, Taphoorn MJB, Janzer RC, et al. Effects of radiotherapy with concomitant and adjuvant temozolomide versus radiotherapy alone on survival in glioblastoma in a randomised phase III study: 5-year analysis of the EORTC-NCIC trial. Lancet Oncol. 2009;10:459–66. doi:10.1016/S1470-2045(09)70025-7.
3. Hochberg FH, Pruitt A. H GBM Path: Assumptions in the radiotherapy of glioblastoma. Neurology. 1980;30:907–11. doi:10.1212/WNL.30.9.907.
4. Stupp R, Brada M, van den Bent MJ, Tonn JC, Pentheroudakis G. High-grade glioma: ESMO clinical practice guidelines for diagnosis, treatment and follow-up. Ann Oncol. 2014;25 January:93–101. doi:10.1093/annonc/mdu050.
5. Bauman G, Fisher B, Watling C, Cairncross JG, Macdonald D. Adult supratentorial low-grade glioma: long-term experience at a single institution. Int J Radiat Oncol Biol Phys. 2009;75:1401–7.
6. Burnet NG, Jefferies SJ, Benson RJ, Hunt DP, Treasure FP. Years of life lost (YLL) from cancer is an important measure of population burden - And should be considered when allocating research funds. Br J Cancer. 2005;92:241–5. doi:10.1038/sj.bjc.6602321.
7. Weller M, Cloughesy T, Perry JR, Wick W. Standards of care for treatment of recurrent. 2012;15:4–27. file:///C:/Users/cpost2/AppData/Local/Mendeley Ltd./Mendeley Desktop/Downloaded/Weller et al. - 2012 - Standards of care for treatment of recurrent.pdf.
8. Neuro-Oncologie LW. Gliomen. Landelijke richtlijn, version 3.0. 2007. file:///C:/Users/cpost2/AppData/Local/Mendeley Ltd./Mendeley Desktop/Downloaded/Landelijke Werkgroep Neuro-Oncologie - 2007 - Gliomen.pdf.
9. Combs SE, Edler L, Rausch R, Welzel T, Wick W, Debus J. Generation and validation of a prognostic score to predict outcome after re-irradiation of recurrent glioma. Acta Oncol (Madr). 2013;52:147–52. doi:10.3109/0284186X.2012.692882.
10. Fogh SE, Andrews DW, Glass J, Curran W, Glass C, Champ C, et al. Hypofractionated stereotactic radiation therapy: An effective therapy for recurrent high-grade gliomas. J Clin Oncol. 2010;28:3048–53. doi:10.1200/JCO.2009.25.6941.
11. Kessel KA, Hesse J, Straube C, Zimmer C, Schmidt-Graf F, Schlegel J, et al. Validation of an established prognostic score after re-irradiation of recurrent glioma Kerstin. Acta Oncol (Madr). 2017;56:422–6. doi:10.1080/0284186X.2016.1276621.
12. Arvold ND, Shi DD, Aizer AA, Norden AD, Reardon DA, Lee EQ, et al. Salvage re-irradiation for recurrent high-grade glioma and comparison to bevacizumab alone. J

Neurooncol. 2017;135:581–91. doi:10.1007/s11060-017-2611-9.
13. Scholtyssek F, Zwiener I, Schlamann A, Seidel C, Meixensberger J, Bauer M, et al. Reirradiation in progressive high-grade gliomas: Outcome, role of concurrent chemotherapy, prognostic factors and validation of a new prognostic score with an independent patient cohort. Radiat Oncol. 2013;8:1–9. doi:10.1186/1748-717X-8-161.
14. Pinzi V, Orsi C, Marchetti M, Milanesi IM, Bianchi LC, DiMeco F, et al. Radiosurgery reirradiation for high-grade glioma recurrence: a retrospective analysis. Neurol Sci. 2015;36:1431–40. doi:10.1007/s10072-015-2172-7.
15. Martinez-Carrillo M, Tovar-Martin I, Zurita-Herrera M, Del Moral-Avila R, Guerrero-Tejada R, Saura-Rojas E, et al. Salvage radiosurgery for selected patients with recurrent malignant gliomas. Biomed Res Int. 2014;2014:657953. doi:10.1155/2014/657953.
16. Patel M, Siddiqui F, Jin JY, Mikkelsen T, Rosenblum M, Movsas B, et al. Salvage reirradiation for recurrent glioblastoma with radiosurgery: Radiographic response and improved survival. J Neurooncol. 2009;92:185–91. doi:10.1007/s11060-008-9752-9.
17. Fokas E, Wacker U, Gross MW, Henzel M, Encheva E, Engenhart-Cabillic R. Hypofractionated Stereotactic Reirradiation of Recurrent Glioblastomas A Beneficial Treatment Option after High-Dose Radiotherapy? Strahlenther Onkol. 2009;185:235–40.
18. Bartsch R, Weitmann HD, Pennwieser W, Wenzel C, Muschitz S, Baldass M, et al. Retrospective analysis of re-irradiation in malignant glioma: A single-center experience. Wien Klin Wochenschr. 2005;117:821–6. doi:10.1007/s00508-005-0475-z.
19. Møller S, Munck af Rosenschöld P, Costa J, Law I, Poulsen HS, Engelholm SA, et al. Toxicity and efficacy of re-irradiation of high-grade glioma in a phase I dose- and volume escalation trial. Radiother Oncol. 2017;125:223–7. doi:10.1016/j.radonc.2017.09.039.
20. Kazmi F, Soon YY, Leong YH, Koh WY, Vellayappan B. Re-irradiation for recurrent glioblastoma (GBM): a systematic review and meta-analysis. J Neurooncol. 2019;142:79–90. doi:10.1007/s11060-018-03064-0.
21. Post CCB, Kramer MCA, Smid EJ, van der Weide HL, Kleynen CE, Heesters MAAM, et al. Patterns of re-irradiation for recurrent gliomas and validation of a prognostic score. Radiother Oncol. doi:10.1016/j.radonc.2018.10.034.
22. Cheng J, Zhang X, Liu B-L. Health-related quality of life in patients with high-grade glioma. Neuro Oncol. 2009;11:41–50. doi:10.1215/15228517-2008-050.
23. Moller S, Law I, Munck af Rosenschold P, Costa J, Poulsen HS, Engelholm SA, et al. Prognostic value of18F-FET PET imaging in re-irradiation of high-grade glioma: Results of a phase I clinical trial. Radiother Oncol. 2016;121:132–7.
24. McKenzie JT, Guarnaschelli JN, Vagal AS, Warnick RE, Breneman JC. Hypofractionated stereotactic radiotherapy for unifocal and multifocal recurrence of malignant gliomas. J Neurooncol. 2013;113:403–9. doi:10.1007/s11060-013-1126-2.

25. Cho KH, Hall WA, Gerbi BJ, Higgins PD, McGuire WA, Clark HB. Single dose versus fractionated stereotactic radiotherapy for recurrent high-grade gliomas. Int J Radiat Oncol Biol Phys. 1999;45:1133–41. doi:10.1016/S0360-3016(99)00336-3.
26. Shepherd SF, Laing RW, Cosgrove VP, Warrington a P, Hines F, Ashley SE, et al. Hypofractionated stereotactic radiotherapy in the management of recurrent glioma. Int J Radiat Oncol Biol Phys. 1997;37:393–8. doi:S0360301696004555 [pii].
27. Shen CJ, Kummerlowe MN, Redmond KJ, Martinez-Gutierrez JC, Usama SM, Holdhoff M, et al. Re-irradiation for malignant glioma: Toward patient selection and defining treatment parameters for salvage. Adv Radiat Oncol. 2018;:1–9. doi:10.1016/J.ADRO.2018.06.005.
28. Lee J, Cho J, Chang JH, Suh CO. Re-irradiation for recurrent gliomas: Treatment outcomes and prognostic factors. Yonsei Med J. 2016;57:824–30. doi:10.3349/ymj.2016.57.4.824.
29. Wen PY, Macdonald DR, Reardon DA, Cloughesy TF, Sorensen AG, Galanis E, et al. Updated response assessment criteria for high-grade gliomas: Response assessment in neuro-oncology working group. J Clin Oncol. 2010;28:1963–72. doi:10.1200/JCO.2009.26.3541.
30. Chow S, Shao J, Wang H. Sample Size Calculations in Clinical Research. Second. Chapman & Hall/CRC Biostatistics Series; 2008.
31. Niyazi M, Adeberg S, Kaul D, Boulesteix AL, Bougatf N, Fleischmann DF, et al. Independent validation of a new reirradiation risk score (RRRS) for glioma patients predicting post-recurrence survival: A multicenter DKTK/ROG analysis. Radiother Oncol. 2018;:1–7. doi:10.1016/j.radonc.2018.01.011.
32. Mayer R, Sminia P. Reirradiation Tolerance of the Human Brain. Int J Radiat Oncol Biol Phys. 2008;70:1350–60. doi:10.1016/j.ijrobp.2007.08.015.
33. Sminia P, Mayer R. External beam radiotherapy of recurrent glioma: Radiation tolerance of the human brain. Cancers (Basel). 2012;4:379–99. doi:10.3390/cancers4020379.
34. Hudes RS, Corn BW, Werner-Wasik M, Andrews D, Rosenstock J, Thoron L, et al. A phase I dose escalation study of hypofractionated stereotactic radiotherapy as salvage therapy for persistent or recurrent malignant glioma. Int J Radiat Oncol Biol Phys. 1999;43:293–8. file:///C:/Users/cpost2/AppData/Local/Mendeley Ltd./Mendeley Desktop/Downloaded/Hudes et al. - 1999 - A phase I dose escalation study of hypofractionated stereotactic radiotherapy as salvage therapy for persistent or.pdf.
35. Barney C, Shukla G, Bhamidipati D, Palmer JD. Re-irradiation for recurrent glioblastoma multiforme. Chinese Clin Oncol. 2017;6:36. doi:10.21037/cco.2017.06.18.
36. Group E. EuroQol-a new facility for the measurement of health-related quality of life. Health Policy (New York). 1990;16:199–208.
37. Herdman M, Gudex C, Lloyd A, Janssen MF, Kind P, Parkin D, et al. Development and

preliminary testing of the new five-level version of EQ-5D (EQ-5D-5L). Qual Life Res. 2011;20:1727–36.
38. Groenvold M, Petersen MA, Aaronson NK, Arraras JI, Blazeby JM, Bottomley A, et al. The development of the EORTC QLQ-C15-PAL: A shortened questionnaire for cancer patients in palliative care. Eur J Cancer. 2006;42:55–64. doi:10.1016/j.ejca.2005.06.022.
39. Caissie A, Nguyen J, Chen E, Zhang L, Sahgal A, Clemons M, et al. Quality of life in patients with brain metastases using the EORTC QLQ-BN20+2 and QLQ-C15-PAL. Int J Radiat Oncol Biol Phys. 2012;83:1238–45. doi:10.1016/j.ijrobp.2011.09.025.
40. Nguyen J, Zhang L, Clemons M, Vassiliou V, Danielson B, Fairchild A, et al. Content validation of the EORTC QLQ-BN20+2 with patients and health care professionals to assess quality of life in brain metastases. J Radiat Oncol. 2012;1:397–409. doi:10.1007/s13566-012-0044-9.
41. Wefel JS, Vardy J, Ahles T, Schagen SB. International Cognition and Cancer Task Force recommendations to harmonise studies of cognitive function in patients with cancer. Lancet Oncol. 2011;12:703–8. doi:10.1016/S1470-2045(10)70294-1.
42. Lin NU, Wefel JS, Lee EQ, Schiff D, van den Bent MJ, Soffietti R, et al. Challenges relating to solid tumour brain metastases in clinical trials, part 2: Neurocognitive, neurological, and quality-of-life outcomes. A report from the RANO group. Lancet Oncol. 2013;14:407–16. doi:10.1016/S1470-2045(13)70308-5.
43. Rehal S, Morris TP, Fielding K, Carpenter JR, Phillips PPJ. Non-inferiority trials: are they inferior? A systematic review of reporting in major medical journals. BMJ Open. 2016;6:e012594–e012594. doi:10.1136/bmjopen-2016-012594.
44. Lee SW, Fraass BA, Marsh LH, Herbort K, Gebarski SS, Martel MK, et al. Patterns of failure following high-dose 3-D conformal radiotherapy for high-grade astrocytomas: A quantitative dosimetric study. Int J Radiat Oncol Biol Phys. 1999;43:79–88.
45. Ivanova A, Qaqish BF, Schell MJ. Continuous toxicity monitoring in phase II trials in oncology. Biometrics. 2005;61:540–5.
46. Brouwer CL, Steenbakkers RJHM, Bourhis J, Budach W, Grau C, Grégoire V, et al. CT-based delineation of organs at risk in the head and neck region: DAHANCA, EORTC, GORTEC, HKNPCSG, NCIC CTG, NCRI, NRG Oncology and TROG consensus guidelines. Radiother Oncol. 2015;117:83–90. doi:10.1016/j.radonc.2015.07.041.
47. Eekers DB, in ‘t Ven L, Roelofs E, et al. The EPTN consensus-based atlas for CT- and MR-based contouring in neuro-oncology. *Radiother Oncol*. 2018;128(1):37-43. doi:10.1016/j.radonc.2017.12.013
48. NIEDER C, ANDRATSCHKE NH, GROSU AL. Re-irradiation for Recurrent Primary Brain Tumors. Anticancer Res. 2016;36:4985–96. doi:10.21873/anticanres.11067.

Appendix

1. Radiotherapy Treatment Protocol for arm 1

1.1 Equipment and Planning
Radiotherapy will be planned and delivered with modern techniques including at least intensity modulated radiation therapy (IMRT) and volumetric modulated arc therapy (VMAT).

1.2 Immobilization
All participants will be immobilized in a customized thermoplastic shell or relocatable stereotactic frame in a supine position according to institutional standards.

1.3 Data acquisition and Definitions of Target Volumes and Organs at Risk
The definition of volumes will be in accordance with the International Commission on Radiation Units and Measurements (ICRU) Report 50, ICRU Report 62. Volumes will be defined based on a volume MRI scan with contrast (T1+Gadolinium, CE-T1) and co-registered to a computerized tomography (CT) scan. The MRI scans must have a maximum 2 mm and isotropic voxels, the CT should have 1-3mm slice thickness. Amino-Acid-PET or SPECT-Examinations may be used in addition to contrast-enhancement on MRI for target volume definition but are not mandatory.

1.3.1 Gross Tumour Volume (GTV)
The GTV is defined as the contrast enhancing lesion on CE-T1 MRI sequence for both treatment groups. GTV can be expanded using the other clinically acquired MRI sequences according to the expertise of the local radiation oncologist and the medical team.

1.3.2 Clinical Target Volume (CTV)
The CTV of both, the experimental arm and the standard arm, is defined as the GTV with addition of a 3-dimensional margin of 5 mm limited by anatomic borders. The stereotactic CTV_{boost} of the experimental arm is equivalent to the GTV. CTV can be expanded using the other clinically acquired MRI sequences according to the expertise of the local radiation oncologist and the medical team.

1.3.3 Planning Target Volume (PTV)
The PTV and PTV_{boost} are defined as the CTV and stereotactic CTV_{boost} plus an isotropic 2 or 3 mm margin depending on radiotherapy center, to account for day to day setup variation related to the ability to immobilize the participant. The PTV should not extend outside the body contour.

1.3.4 Organs at Risk (OAR)
OAR include the following normal tissue volumes: the lenses, optic nerves and chiasm and the eyeball (includes anterior and posterior chambers); brainstem; pituitary; cochlea; skin (a 3 mm thick rim) and the uninvolved brain (= brain – CTV). OARs will be delineated following the local guidelines of the participating centers and will typically follow atlases such as CT-based OAR atlas [46] or the European Partical Therapy Network (EPTN)-consensus based atlas [47]summarized in Table A1. The complete atlas is provided as supplemental material

of the published article, available at https://ars.els-cdn.com/content/image/1-s2.0-S0167814015004016-mmc1.pdf .

Table A1. Definition of OARs summarized from the head and neck OAR guideline (DAHANCA, EORTC, GORTEC, HKNPCSG, NCIC CTG, NCRI, NRG Oncology and TROG consensus guidelines) [46].

**Organ**	**Definition**
Eyeball	The anterior and posterior segment of the eyeball are combined.<br>The anterior segment of the eyeball consists of the structures ventral from the vitreous humor, including the cornea, iris, ciliary body, and lens.<br>The posterior segment of the eyeball is located posteriorly to the lens, and consists of the anterior hyaloid membrane and all of the posterior optical structures including the vitreous humor, retina, and choroid. The optic nerve is excluded from this contour. The entire retina is included in the posterior segment of the eyeball.
Cochlea	The cochlea is embedded in the temporal bone, located lateral to the internal auditory meatus, which can best be recognized in CT bone view settings
Brainstem	The cranial border of the brainstem is defined as the slice where the optic chiasm is visible, the caudal border as the tip of the dens of C2 (cranial border of the spinal cord). MRI is recommended for delineation of the brainstem.
Pituitary gland	The pituitary gland is a very small OAR, which in general cannot be identified easily on CT. Alternatively, therefore, the inner part of the sella turcica will be used as surrogate anatomical bony structure.<br>The borders of the pituitary gland can be defined best in the sagittal view.
Optic chiasm	The optic chiasm is located in the subarachnoid space of the suprasellar cistern. Typically, it is located 1 cm superior to the pituitary gland, located in the sella turcica. MRI is recommended for delineation of the optic chiasm, 5mm of optic nerve and tract is incorporated in the chiasm, forming an X.
Optic nerve	The optic nerve is usually 2–5 mm thick and in general is clearly identifiable on CT. It has to be contoured all the way from the posterior edge of the eyeball, through the bony optic canal to the optic chiasm. MRI is recommended for a better delineation of the optic nerve, at least close to the optic chiasm.
Brain	The delineation of the brain includes brain vessels, and excludes the brainstem. CT bone settings are recommended.

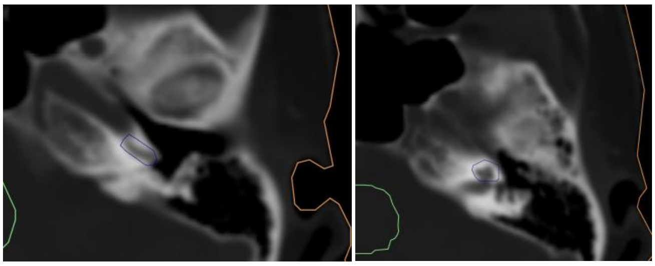

Figure A1. Delineation of the cochlea in CT bone settings (purple).

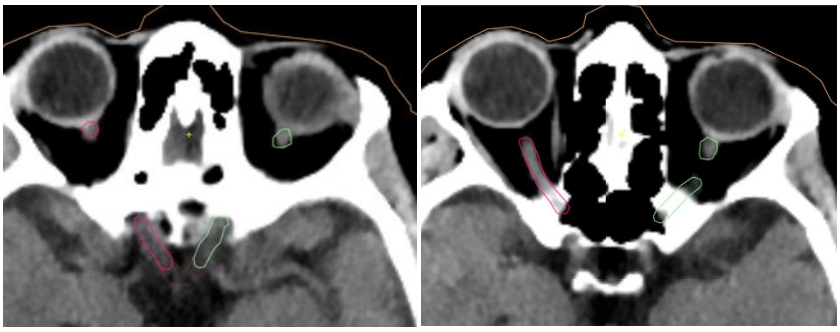

Figure A2. Delineation of the optic nerves (pink and green).

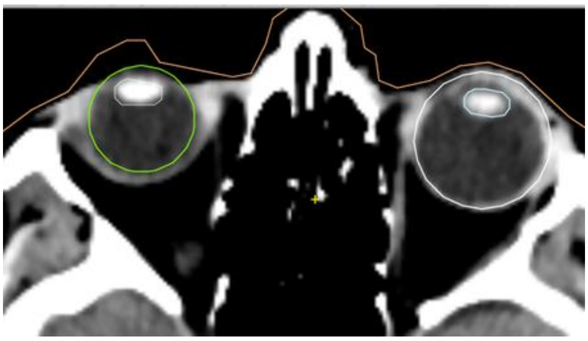

Figure A3. Delineation of the eyeballs (green, white) and lenses (white).

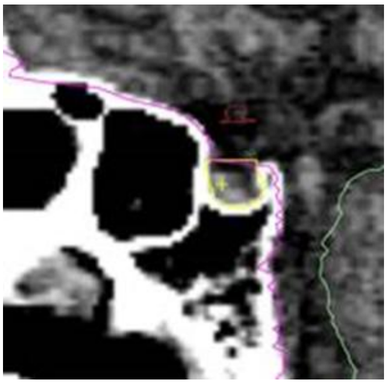

Figure A4. Delineation of the pituitary gland (yellow) in CT bone setting.

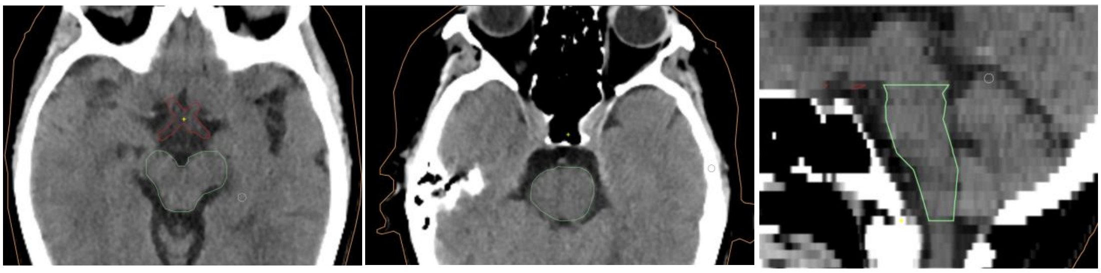

Figure A5. Delineation of the optic chiasm (brown) and brainstem (green).

1.3.5 Recovery potential

With regard to the recovery potential in the brain after prior irradiation, the following schedule will be utilized[48]:

- No recovery within 1 year.
- 30% recovery after 1 year.
- 50% recovery after 2 years.

1.4 Treatment planning

IMRT or VMAT may be planned using fixed fields or arcs. The treatment plan to be used for each participant is based on an analysis of volumetric doses including the dose volume histogram (DVH) of the PTV and the OARs. Treatment planning should conform to ICRU 50, 62 and 83 rules for coverage of GTV, CTV and PTV. Additionally, organs at risk (OAR) detailed in Appendix chapter 1.3.4 should be delineated according to the ICRU 62 rules.

OAR planning risk volumes (PRV) should be defined by applying a margin equal to the PTV margin.

1.5 Prescribed dose and fractionation

The dose prescribed to the PTV in the experimental arm is 4 fractions of 5.5 Gy. The prescribed dose to the PTV_{boost} is 4 fractions of 7.5 Gy. All fractions should be given within 2 weeks. The maximum dose in the PTV_{boost} of the experimental arm should not exceed 130% of the prescription dose. Maximum dose in the PTV should not exceed 107% of the prescription dose according to ICRU rapport 50. The dose calculation grid should be 3 mm or less.

1.6 Dose Limitation to Organs at Risk

It is important to minimize the dose to the OARs whenever possible. This must be weighed against the possibility of sub-optimal treatment of the target volume.

Table A1. Dose constraints for OARs in Gy. Radiobiological calculations performed with α/β = 2Gy. To calculate the dose constraint at ReRT, the initial dose should be subtracted from the cumulative dose constraint for OARs. Subsequently, the ReRT EQD2 should be converted to the dose constraint for the OARs according to the treatment arm. The ReRT dose of OARs should never exceed the EQD2 and total dose constraint given for initial treatment (shown in column 'Constraint at ReRT').
Example: Optic Chiasm ReRT dose = 9.8Gy (≤ 38) in 10 fractions = 7Gy EQD2 (≤ 54). Initial dose 53.2Gy in 30 fractions = 51Gy EQD2. Cumulative dose = 51 + 7 = 58Gy, is ≤ 81.

Constraint	OAR	Constraint at ReRT (without considering previous radiation therapy)		Cumulative constraint (Initial RT + ReRT)
		Total $D_{max\ 0.1cc}$ dose (unless otherwise specified) at **α/β=2**		
		EQD2	Total physical dose at 4 x 7.5 Gy and 4 x 5.5 Gy	EQD2
Hard	Optic nerves	≤ 60 Gy	≤ 27 Gy	≤ 90 Gy
	Optic chiasm	≤ 60 Gy	≤ 27 Gy	≤ 90 Gy
	Brain stem	≤ 60 Gy	≤ 27 Gy	≤ 90 Gy
Soft	Optic nerves	≤ 55 Gy	≤ 26 Gy	≤ 83 Gy
	Optic chiasm	≤ 55 Gy	≤ 26 Gy	≤ 83 Gy
	Brain stem	≤ 55 Gy OR 54-59 Gy < 10cc	≤ 26 Gy OR 26-27 Gy < 10cc	≤ 83 Gy OR 81-89 Gy < 10 cc
	Eye balls	≤ 45	≤ 23 Gy	≤ 68 Gy
	Lenses	D_{Mean} ≤6 Gy D_{max} 10 Gy	D_{Mean} ≤6 Gy $D_{max\ 0.1cc}$ 9 Gy	D_{Mean} ≤9 Gy $D_{max\ 0.1cc}$ 15 Gy
	Pituitary Gland	D_{Mean} ≤ 45 Gy	D_{Mean} ≤ 23 Gy	D_{Mean} ≤ 68 Gy
	Cochlea	D_{Mean} ≤ 45 Gy	D_{Mean} ≤ 23 Gy	D_{Mean} ≤ 68 Gy

1.7 Timing of radiotherapy

The interval between the planning-MRI and actual first fraction of ReRT is 3 weeks at most.

1.8 Treatment Protocol Overview Table

Table A2. Overview of target volume definitions, dose prescription and accepted dose heterogeneity. *Depending on radiotherapy center

	**Experimental arm**<br>**4 x 5.5 Gy**<br>**with 7.5 Gy boost**
**GTV**	Contrast enhancement on T1 MRI
**$CTV_{30Gy\ (boost)}$**	GTV
**CTV_{22Gy}**	GTV + 5 mm
**$PTV_{30Gy\ (boost)}$**	$CTV_{30Gy\ (boost)}$ + 2 or 3 mm
**PTV_{22Gy}**	CTV_{22Gy} + 2 or 3 mm
**Accepted dose heterogeneity $PTV_{30Gy\ (boost)}$**	95% - 107%<br>$D_{max\ 0.1cc}$
**Accepted dose heterogeneity PTV_{22Gy}**	95% - 130% (exclude PTV_{36Gy})<br>$D_{max\ 0.1cc}$

2. Toxicity assessed by CTCAE v5.0

Toxicity assessment for outcome registration and analysis. NB: SAE is not assessed by CTCAE, for serious adverse events description see chapter 7.2.

	1	2	3	4	5
Central nervous system necrosis	Asymptomatic; clinical or diagnostic observations only; intervention not indicated	Moderate symptoms; corticosteroids indicated	Severe symptoms; medical intervention indicated	Life-threatening consequences; urgent intervention indicated	Death
Headache	Mild pain	Moderate pain; limiting instrumental ADL	Severe pain; limiting self-care ADL	-	-
Seizure	Brief partial seizure and no loss of consciousness	Brief generalized seizure	New onset seizures (partial or generalized); multiple seizures despite medical intervention	Life-threatening consequences; prolonged repetitive seizures	Death
Fatigue	Fatigue relieved by rest	Fatigue not relieved by rest; limiting instrumental ADL	Fatigue not relieved by rest, limiting self-care ADL	-	-
Nausea	Loss of appetite without alteration in eating habits	Oral intake decreased without significant weight loss, dehydration or malnutrition	Inadequate oral caloric or fluid intake; tube feeding, TPN, or hospitalization indicated	-	-
Dermatitis radiation	Faint erythema or dry desquamation	Moderate to brisk erythema; patchy moist desquamation, mostly confined to skin folds and creases; moderate edema	Moist desquamation in areas other than skin folds and creases; bleeding induced by minor trauma or abrasion	Life-threatening consequences; skin necrosis or ulceration of full thickness dermis; spontaneous bleeding from involved site; skin graft indicated	Death

Nervous system disorders - Other, specify	Asymptomatic or mild symptoms; clinical or diagnostic observations only; intervention not indicated	Moderate; minimal, local or noninvasive intervention indicated; limiting age-appropriate instrumental ADL	Severe or medically significant but not immediately life-threatening; hospitalization or prolongation of existing hospitalization indicated; limiting self-care ADL	Life-threatening consequences; urgent intervention indicated	Death

Hospitalization (or prolongation of hospitalization):
The National Cancer Institute defines hospitalization for expedited AE reporting purposes as an inpatient hospital stay equal to or greater than 24 hours. Hospitalization is used as an indicator of the seriousness of the AE and should ONLY be used for situations where the AE truly fits this definition and NOT for hospitalizations associated with less serious events. (*e.g.*, a hospital visit where a patient is admitted for observation or minor treatment such as, hydration and released in less than 24 hours). Furthermore, hospitalization for pharmacokinetic sampling is not an AE, and therefore is not to be reported either as a routine AE or in an expedited report.

Attribution
An assessment of the relationship between the AE and the medical intervention. CTCAE does not define an AE as necessarily “caused by a therapeutic intervention”. After naming and grading the event, the clinical investigator must assign an attribution to the AE using the following attribution categories:

Unrelated to investigational agent/intervention	
Unrelated	The AE *is clearly NOT related* to the intervention
Unlikely	The AE *is doubtfully related* to the intervention
Related to investigational agent/intervention	
Possible	The AE *may be related* to the intervention
Probable	The AE *is likely related* to the Intervention
Definite	The AE *is clearly related* to the intervention
